# Chemical Analysis of Pollen by FT-Raman and FTIR Spectroscopies

**DOI:** 10.3389/fpls.2020.00352

**Published:** 2020-03-31

**Authors:** Adriana Kenđel, Boris Zimmermann

**Affiliations:** ^1^Division of Analytical Chemistry, Department of Chemistry, Faculty of Science, University of Zagreb, Zagreb, Croatia; ^2^Faculty of Science and Technology, Norwegian University of Life Sciences, Ås, Norway; ^3^Division of Organic Chemistry and Biochemistry, Ruđer Bošković Institute, Zagreb, Croatia

**Keywords:** Raman spectroscopy, Fourier transform infrared spectroscopy, multivariate analysis, male gametophyte, flowering, pollen wall, pollination, palynology

## Abstract

Pollen studies are important for the assessment of present and past environment, including biodiversity, sexual reproduction of plants and plant-pollinator interactions, monitoring of aeroallergens, and impact of climate and pollution on wild communities and cultivated crops. Although information on chemical composition of pollen is of importance in all of those research areas, pollen chemistry has been rarely measured due to complex and time-consuming analyses. Vibrational spectroscopies, coupled with multivariate data analysis, have shown great potential for rapid chemical characterization, identification and classification of pollen. This study, comprising 219 species from all principal taxa of seed plants, has demonstrated that high-quality Raman spectra of pollen can be obtained by Fourier transform (FT) Raman spectroscopy. In combination with Fourier transform infrared spectroscopy (FTIR), FT-Raman spectroscopy is obtaining comprehensive information on pollen chemistry. Presence of all the main biochemical constituents of pollen, such as proteins, lipids, carbohydrates, carotenoids and sporopollenins, have been identified and detected in the spectra, and the study shows approaches to measure relative and absolute content of these constituents. The results show that FT-Raman spectroscopy has clear advantage over standard dispersive Raman measurements, in particular for measurement of pollen samples with high pigment content. FT-Raman spectra are strongly biased toward chemical composition of pollen wall constituents, namely sporopollenins and pigments. This makes Raman spectra complementary to FTIR spectra, which over-represent chemical constituents of the grain interior, such as lipids and carbohydrates. The results show a large variability in pollen chemistry for families, genera and even congeneric species, revealing wide range of reproductive strategies, from storage of nutrients to variation in carotenoids and phenylpropanoids. The information on pollen’s chemical patterns for major plant taxa should be of outstanding value for various studies in plant biology and ecology, including aerobiology, palaeoecology, forensics, community ecology, plant-pollinator interactions, and climate effects on plants.

## Introduction

Pollen is multicellular haploid gametophyte life stage of seed plants (spermatophytes) and thus it has a key function in plant life cycle. Due to their high mobility by abiotic and biotic pollination vectors, pollen play an essential role in the gene flow within and among plant populations. Therefore, pollen studies are important for assessment of environment, including biodiversity, plant–pollinator interactions, and impact of climate and pollution on wild communities and cultivated crops. Moreover, pollen is seasonal air pollutant that can trigger allergy-related respiratory diseases, and thus pollen monitoring is needed for avoidance and timely treatment of symptoms. Finally, fossil pollen grains are often the most abundant and the best preserved remains of plant species, thus providing crucial information for the reconstruction of past terrestrial communities and climate conditions (Lindbladh et al., 2002; Jardine et al., 2016). In general, pollen studies can provide information on spatial and temporal distribution of organisms and populations, as well as on the biological and environmental processes influencing them. As a result, pollen studies have been extensively conducted in biology, ecology, palaeoecology, medicine, agronomy, and forensics.

Most of the pollen studies are focused on a quite limited number of traits, such as pollen morphology, pollen production per flower, pollen transfer, pollinator attraction, and pollen viability (Bell, 1959; Bassani et al., 1994; Molina et al., 1996; Pacini et al., 1997; Streiff et al., 1999; Tamura and Kudo, 2000; Oddou-Muratorio et al., 2005; Hall and Walter, 2011; Welsford et al., 2016). The most important reason for such deficiency of data, compared to information on female traits, is relative difficulty of quantitative and qualitative measurements of male traits (Williams and Mazer, 2016). In particular, pollen chemical composition has been rarely measured due to complex and time-consuming analyses. For example, triglyceride lipids (triacylglycerols) primarily serve as carbon and long-term energy reserves in a form of lipid bodies that play a crucial role, as a source of materials and energy, in germination of pollen as well as in pollen tube growth (Piffanelli et al., 1998; Rodriguez-Garcia et al., 2003). This is of importance since the reproduction of seed plants involves competition among growing pollen tubes to reach and penetrate the ovule. Carbohydrates, in the form of cytoplasmic saccharides, have a vital function in the resistance of pollen to dehydration and temperature stress, as well as serving as grain wall components (cellulose) and energy reserves for germination (starch and sucrose) (Pacini et al., 2006; Bokszczanin et al., 2013). Pollen’s proteins have both structural and functional role, and have implication for both pollen-pistil and plant-pollinator interactions (Roulston et al., 2000). Pollen proteins are important source of dietary nitrogen for a majority of pollinators, while as enzymes they have crucial function during pollen tube growth (Roulston et al., 2000). Pigments, such as carotenoids and flavonoids, participate in light harvesting, serve as cellular membrane protectants from photooxidative damage, and as pathogen defense (Fambrini et al., 2010; Lutz, 2010). Finally, sporopollenins are complex and resilient grain wall biopolymers that protect the grain interior from environmental effects (Li et al., 2019), and can provide valuable information on past environmental conditions (Lomax et al., 2012).

Vibrational spectroscopy of pollen offers a novel approach in plant phenomics via precise and comprehensive measurement of pollen’s biochemical ‘fingerprint.’ Vibrational spectra of pollen contain specific signals of lipids, proteins, carbohydrates and water, and even some minor biochemical constituents, such as pigments, can be precisely measured (Schulte et al., 2008, 2009; Zimmermann, 2010; Pummer et al., 2013; Zimmermann and Kohler, 2014; Bagcioglu et al., 2015; Jardine et al., 2015). The compounds that are measurable by vibrational spectroscopy are the principal structural and nutritious components, and they are responsible for the majority of chemical phenotypic attributes of pollen. Therefore, chemical analysis of pollen by vibrational spectroscopy offers complementary information to the contemporary ‘omics-based’ approaches, such as genomics and transcriptomics by sequencing technologies, as well as to proteomics and metabolomics by mass spectrometry and NMR spectroscopy.

Vibrational studies of pollen, by diverse infrared and Raman techniques, have shown that vibrational spectroscopy achieves economical and rapid identification and classification of pollen according to taxonomy and phylogenetic relationship (Pappas et al., 2003; Ivleva et al., 2005; Gottardini et al., 2007; Schulte et al., 2008; Dell’anna et al., 2009; Zimmermann, 2010, 2018; Guedes et al., 2014; Zimmermann and Kohler, 2014; Zimmermann et al., 2015b; Julier et al., 2016; Seifert et al., 2016; Woutersen et al., 2018; Jardine et al., 2019; Mondol et al., 2019). For example, the recent FTIR microspectroscopy study on individual pollen grains has achieved more accurate classification than optical microscopy, which is the benchmark method in pollen identification (Zimmermann et al., 2016). Moreover, FTIR microspectroscopy enables chemical imaging of pollen grain ultrastructure (Zimmermann et al., 2015a), while even higher spatial resolution of Raman microspectroscopy enables monitoring of the molecular composition during pollen germination and pollen tube growth (Schulte et al., 2010; Joester et al., 2017). In addition to classification and identification studies, vibrational spectroscopies provide biochemical characterization of pollen with respect to environmental conditions. For example, differences in chemical phenotypes of pollen were measured with respect to nutrient availability (Zimmermann et al., 2017), heat stress (Lahlali et al., 2014; Jiang et al., 2015), pollution stress (Depciuch et al., 2016, 2017), location (Bağcıoğlu et al., 2017; Zimmermann et al., 2017), and season (Zimmermann and Kohler, 2014; Bağcıoğlu et al., 2017). In general, Raman and Fourier transform infrared (FTIR) spectroscopies provide chemically complementary information, and therefore measurement of samples by both techniques provides highly detailed biochemical characterization (Zimmermann, 2010; Pummer et al., 2013; Bagcioglu et al., 2015; Zimmermann et al., 2015a; Diehn et al., 2020).

One important advantage of Raman spectroscopy of pollen over FTIR approach is the obtained information on grain wall pigments, in particular carotenoids, which cannot be measured at all by FTIR due to low concentrations and weak signals (Schulte et al., 2009). Unfortunately, Raman measurements are often hindered by laser-induced degradation (burning) of pollen grains, and by the strong fluorescence background that often masks any underlying Raman spectra (Ivleva et al., 2005; Schulte et al., 2008; Guedes et al., 2014). An interesting aspect of Raman spectroscopy is that pigments, such as carotenoids and chlorophylls, exhibit resonance Raman spectra of various intensities (De Oliveira et al., 2009). Resonance Raman spectra occur when the wavelength of the excitation laser coincides with electronic transition. For example, the conjugated nature of π-electrons from the polyene backbone of carotenoids results in electronic states of lower energy, often with absorption in the visible part of the spectrum. For this reason, carotenoids usually display strong yellow, orange and red colors. Moreover, this can cause strong enhancement of vibrational bands in carotenoids, especially those at 1530 (related to −C=C− bonds) and 1160 cm^–1^ (related to −C−C− bonds) that have strong electron-phonon coupling. Resonance Raman spectra of pigments enable measurement of very low concentration of pigments (Schulte et al., 2009). However, resonant Raman spectra can also mask completely the regular Raman spectral contributions from other compounds, thus hindering their analysis (Schulte et al., 2008). Use of visible excitation lasers, such as 633 nm, often results with strong light absorbance of pollen sample, leading to sample heating and even photodegradation (Schulte et al., 2008, 2009).

These problems, that are common in conventional (dispersive) Raman measurements, can be addressed by Fourier transform (FT) Raman spectroscopy that uses high-wavelength near infrared (NIR) laser excitation. In general, electronic transitions are weaker at longer wavelengths, and thus detrimental effect of sample heating can be avoided by use of NIR lasers. Moreover, the frequency of the NIR laser usually does not correspond to an electronic transition of the sample, thus diminishing possibility for the occurrence of fluorescence. Finally, use of longer Raman excitation wavelengths can significantly increase penetration depth, compared to short-wavelength lasers, thus more comprehensive information on pollen composition could be obtained (Moester et al., 2019). However, these important advantages of FT-Raman spectrometers can be overshadowed by sensitivity advantage of dispersive Raman spectrometers with short-wavelength laser excitation. Nevertheless, our preliminary study, employing FT-Raman spectroscopy for measurement of 43 conifer species, has shown that high-quality FT-Raman spectra of pollen can be recorded (Zimmermann, 2010). The spectra were devoid of detrimental fluorescence and heating effects, thus indicating great potential of FT-Raman spectroscopy for identification and analysis of plants.

In the paper at hand, we explore the use of FT-Raman and FTIR spectroscopy for chemical characterization of pollen. The study was conducted on a diverse set of plants, comprising 219 species, belonging to 42 families, and covering all major taxa of seed plants. Firstly, we wanted to demonstrate that high-quality Raman spectra of pollen can be obtained by FT-Raman technique. In particular, Raman spectra of pollen samples with high pigment content, which regularly cannot be measured intact with dispersive Raman, were obtained by FT-Raman spectroscopy. Secondly, the study highlights unique pollen chemistry information obtained by either FT-Raman or FTIR approach, and thus demonstrates advantages of the combined approach with both techniques. The biochemical characterization of pollen for the major plant lineages is provided, in particular regarding lipid, protein, carbohydrate, carotenoid and phenylpropanoid content. Pollen chemistry, obtained by the spectroscopic approach, was discussed in relation to the results of the standard chemical analysis pollen studies. Moreover, quantitative measurement of pollen protein content has been provided by combining spectroscopy and chemometrics, clearly demonstrating potential of vibrational spectroscopy for not only qualitative but also quantitative chemical analysis of pollen.

## Materials and Methods

### Samples

Samples of pollen were collected at two facilities of the University of Zagreb; the Botanical Garden of the Faculty of Science and the Botanical Garden “Fran Kušan” of the Faculty of Pharmacy and Biochemistry. Both locations are situated within 1.5 km radius and can be considered the same climate area. 219 samples were collected altogether, each belonging to different plant species (Table 1, Supplementary Table S1, and Supplementary Figure S1). Pollen samples were collected during 2011 and 2012 pollination seasons. The pollen samples were collected directly from plants at flowering time, either by shaking flowers (anemophilous species) or collecting mature anthers (entomophilous species). Only one sample per species was created, either by collecting pollen from only one plant or by collecting pollen from several individuals of the same species followed by merging of all the collected pollen into one sample. The samples were kept in paper bags at r.t. for 24 h (together with anthers for entomophilous species), and afterward transferred to vials as dry powder and stored at −15°C. For the spectroscopic measurements, three replicates per technique were measured, each replicate comprising approx. 0.5–1.0 mg of pollen sample. Approx. 10^3^–10^5^ pollen grains per replicate were measured, and considering that each pollen grain has a unique genotype (and implicitly phenotype), each measurement comprised biologically distinct pollen population. However, in a majority of cases (for example for all tree species) genetic pool was very limited since it originated from the same sporophyte parent plant. Therefore, the presented variation in the spectral sets can be considered a preliminary estimate for the measured plant groups. Influence of larger genetic pools, growth conditions, location and year of pollination on pollen chemical composition and pollen classification for a number of plant groups (e.g., grasses, pines, and oaks) were covered in our previous studies (Zimmermann and Kohler, 2014; Bağcıoğlu et al., 2017; Zimmermann et al., 2017; Diehn et al., 2020).

**TABLE 1:** List of analyzed plant taxa with number of genera and species covered by the study (see Supplementary Table S1 for details).

Clade	Order	Family	No. of genera	No. of species
Eudicots	Fagales	Betulaceae	5	13
		Fagaceae	2	11
		Juglandaceae	3	6
	Malpighiales	Salicaceae	2	3
		Euphorbiaceae	1	1
	Lamiales	Oleaceae	1	3
		Plantaginaceae	2	4
		Scrophulariaceae	1	1
		Acanthaceae	1	1
	Solanales	Solanaceae	3	4
	Proteales	Platanaceae	1	2
	Saxifragales	Altingiaceae	1	2
		Paeoniaceae	1	2
	Sapindales	Sapindaceae	1	1
		Anacardiaceae	1	2
		Rutaceae	1	1
	Dipsacales	Adoxaceae	2	3
		Dipsacaceae	1	1
	Buxales	Buxaceae	1	2
	Rosales	Rosaceae	4	5
		Urticaceae	1	1
	Ranunculales	Ranunculaceae	5	10
		Papaveraceae	3	5
	Caryophyllales	Polygonaceae	1	4
	Asterales	Asteraceae	2	3
		Campanulaceae	1	1
	Malvales	Malvaceae	3	3
Magnoliids	Magnoliales	Magnoliaceae	2	2
Monocots	Poales	Cyperaceae	3	11
		Poaceae	9	18
		Juncaceae	1	2
	Asparagales	Iridaceae	1	17
		Xanthorrhoeaceae	2	4
	Liliales	Liliaceae	2	10
	Arecales	Arecaceae	1	1
Gymnosperms	Pinales	Cupressaceae	11	23
		Taxaceae	1	2
		Cephalotaxaceae	2	2
		Pinaceae	5	29
		Podocarpaceae	1	1
	Ginkgoales	Ginkgoaceae	1	1
	Ephedrales	Ephedraceae	1	1

For identification of basic biochemicals in pollen a set of model compounds was measured to correlate with high positive or negative values in the principal component analyses loadings plots. Spectra of crystal lipids and carbohydrates were recorded above their melting temperature, and again at r.t. after cooling to obtain spectrum of amorphous phase (liquid and/or glass phase). Lipids: Tristearin (2,3-di(octadecanoyloxy)propyl octadecanoate), triolein (2,3-bis[[(*Z*)-octadec-9-enoyl]oxy]propyl (*Z*)-octadec-9-enoate), tri- heptadecanoin (2,3-di(heptadecanoyloxy)propyl heptadeca- noate), phosphatidistearoylcholine (1,2-distearoyl-rac-glycero- 3-phosphocholine), phosphatidioleylcholine (1,2-dioleoyl-sn-glycero-3-phosphocholine), stearic acid (octadecanoic acid), oleic acid ((9Z)-octadec- 9-enoic acid). Pigments and phenylpropanoids: rutin, β-carotene, *p*-coumaric acid, ferulic acid, caffeic acid, sinapic acid, hydro-*p*-coumaric acid, hydroferulic acid, hydrocaffeic acid. Carbohydrates: cellulose, amylose, amylopectin, arabinoxylan, pectin, β-D-glucan, sucrose, trehalose, fructose, glucose. Proteins: gluten. All chemicals were purchased from Merck (Darmstadt, Germany) and Sigma-Aldrich (St. Louis, United States), and used without further purification.

### Spectroscopic Analyses

The Raman spectra in backscattering geometry were recorded on a FT-Raman FRA 106/S model, coupled with a Bruker Equinox 55 IR spectrometer, equipped with a neodymium-doped yttrium aluminum garnet (Nd:YAG) laser (1064 nm, 9394 cm^–1^), and germanium detector cooled with liquid nitrogen. The spectra were recorded with a resolution of 4 cm^–1^, with a digital resolution of 1.9 cm^–1^, and with a total of 64 scans, using Blackman–Harris 4- term apodization and with a laser power of 400 mW. Each pollen sample was measured in three replicates.

The infrared spectra were recorded on an ABB Bomem (Quebec City, Canada) MB102 single-beam spectrometer, equipped with cesium iodide optics and deuterated triglycine sulfate (DTGS) detector. The reflectance spectra were recorded by using the single-reflection attenuated total reflectance (SR-ATR) accessory with the horizontal diamond prism and with 45° angle of incidence. The SR-ATR infrared spectra were measured with a Specac (Slough, United Kingdom) Golden Gate ATR Mk II or a Specac High Temperature Golden Gate ATR Mk II. The spectra were recorded with a spectral resolution of 4 cm^–1^, with a digital resolution of 1.9 cm^–1^, and with a total of 30 scans, using cosine apodization. Each spectrum was recorded as the ratio of the sample spectrum to the spectrum of the empty ATR plate. Each pollen sample was measured in three replicates.

### Spectral Pre-processing and Data Analysis

The spectra were pre-processed prior to calibration: all spectra were smoothed by the Savitzky–Golay algorithm using a polynomial of degree two and a window size of 11 points in total, followed by normalization by extended multiplicative signal correction (EMSC), an MSC model extended by a linear and quadratic component (Zimmermann and Kohler, 2013; Guo et al., 2018). The following spectral regions were selected for data analysis: 1900-800 cm^–1^ for infrared spectra, and 2000-500 cm^–1^ for Raman spectra. In the EMSC pre-processing, the spectral region of chemical absorbance was down-weighted, and spectral regions devoid of any chemical absorbance were up-weighted, by applying a weighting vector. Vector value 1 was used in the whole spectral region, except the regions 1900-1800 cm^–1^ (for IR spectra) and 2000-1800 cm^–1^ (for Raman spectra), where the weighting vector was set to 10. These two regions are devoid of any chemical signals. Therefore, these regions should have the same baseline values in all pollen spectra when interferent signals, due to light reflection or fluorescence, have been removed. Up-weighting of this region, by applying a weighting vector, is constraining the EMSC pre-processing and ensuring a stable baseline in all pollen spectra. Thus pre-processed spectra were designated *Datasets I* and subsequently used to evaluate biochemical similarities between pollen samples by calculating correlation coefficients (Pearson product-moment correlation coefficients) or by using principal component analysis (PCA). For better viewing the figures depicting correlation matrixes and PCA plots were based on averaged spectra, where spectra of 3 replicates were averaged.

The estimates of relative chemical composition of pollen were obtained by deflating the data matrix containing complete set of spectra from *Datasets I* by using spectra of standard compounds (Zimmermann and Kohler, 2014). In general, matrix deflation modifies a data matrix to eliminate the influence of a given eigenvector (White, 1958). However, here we used model compounds as eigenvectors while the corresponding eigenvalues were used to estimate the relative content of those compounds. For the deflation, the data matrix was centered while the vectors were normalized. Tristearin, gluten and amylose FTIR spectra were used as eigenvectors for FTIR dataset to estimate relative amounts of triglicerides, proteins and carbohydrates respectively. β-carotene and amylose FT-Raman spectra were used as eigenvectors for Raman dataset to estimate relative amounts of carotenoids and carbohydrates respectively. The corresponding eigenvalues, as well as ratios of eigenvalues (carbohydrate-to-protein ration), were plotted in order to visualize chemical composition of pollen.

*Datasets I* were used for the analysis of pollination strategy by denoting the following taxa: (1) anemophilous: Fagales (except Fagaceae), Pinales (except Podocarpaceae), Poales, Proteales, Anacardiaceae, Asteraceae (except *Taraxacum*), Polygonaceae, Urticaceae, *Plantago*; (2) entomophilous: Asparagales, Dipsacales, Liliales, Magnoliales, Malvales, Ranunculales, Sapindales (except Anacardiaceae), Solanales, Acanthaceae, Campanulaceae, Paeoniaceae, Rosaceae, Scrophulariaceae, *Digitalis*, *Taraxacum*; (3) double-strategy: Arecales, Buxales, Ephedrales, Malpighiales, Altingiaceae, Fagaceae, Ginkgoaceae, Oleaceae, Podocarpaceae.

The protein content of pollen from Roulston et al. (2000) was used as a chemical reference values for regression in the Partial Least Squares Regression (PLSR) modeling of spectral data from *Datasets I*, where spectra of replicates were averaged. The optimal number of components (i.e., PLSR factors) of the calibration models (*A*_*Opt*_) was determined using full cross-validation. The PLSR coefficient of determination (R^2^), correlation value (R), and root-mean-square error (RMSE) were used to evaluate the calibration models. The following 35 species were included in the PLSR: *Fagus sylvatica, Quercus rubra, Quercus robur, Corylus avellana, Alnus incana, Alnus glutinosa, Betula pendula, Juglans nigra, Juglans regia, Carya illinoinensis, Zea mays, Secale cereale, Festuca pratensis, Poa pratensis, Poa nemoralis, Dactylis glomerata, Holcus lanatus, Juniperus communis, Thuja occidentalis, Picea abies, Pinus mugo, Pinus sylvestris, Pinus ponderosa, Eschscholzia californica, Magnolia x sonlangiana, Liriodendron tulipifera, Fraxinus excelsior, Plantago lanceolata, Salix alba, Taraxacum officinale, Populus nigra, Aesculus hippocastanum, Buxus sempervirens, Artemisia vulgaris, Rumex acetosa*.

The following spectral regions were selected for analysis of chemical composition of aromatics in pollen grain wall: 810 – 860 cm^–1^ for FTIR spectra, and 1580 – 1650 cm^–1^ for FT-Raman spectra. Prior to the selection of spectral regions, the EMSC pre-processing was conducted by applying a weighting vector: Vector value 1 was used in the whole spectral region, except the regions 1900-1800 cm^–1^ (for IR spectra) and 1800-1660 cm^–1^ (for FT-Raman spectra), where the weighting vector was set to 10. Thus pre-processed spectra were designated *Datasets II* and subsequently analyzed by PCA.

All pre-processing methods and data analyses were performed using The Unscrambler X 10.3 (CAMO Software, Oslo, Norway), as well as functions and in-house developed routines written in MATLAB 2014a.8.3.0.532 (The MathWorks, Natick, MA, United States).

## Results and Discussion

### Vibrational Spectra of Pollen

**FIGURE 1:**
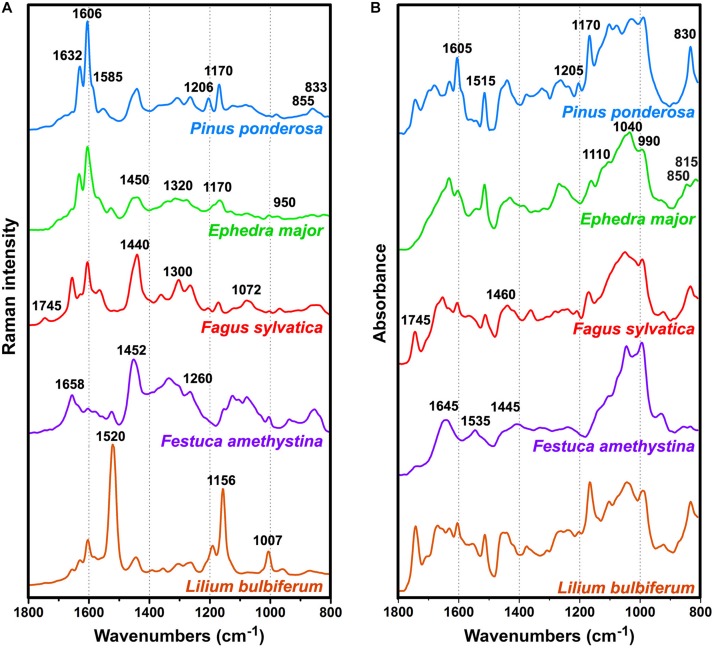
Pre-processed **(A)** FT-Raman and **(B)** FTIR spectra of representative species: *Pinus ponderosa* (with denoted sporopollenin bands), *Ephedra major* (with denoted carbohydrate signals), *Fagus sylvatica* (with denoted lipid signals), *Festuca amethystina* (with denoted protein signals), and *Lilium bulbiferum* (with denoted carotenoid signals).

As mentioned in the Introduction, the major problem in Raman spectroscopy of pollen is sample heating and fluorescence, resulting with complex background and low signal-to-noise ratio. In contrast, the spectra of all 219 pollen samples covered by this study are devoid of strong fluorescence background and have high signal-to-noise ratio (Supplementary Figure S2). The vibrational spectra of pollen of representative species show influence of different biochemicals on an overall spectral fingerprint (Figure 1) (Gottardini et al., 2007; Schulte et al., 2008; Zimmermann, 2010; Bagcioglu et al., 2015; Zimmermann et al., 2015b). In some species, such as gymnosperms (e.g., Figure 1 *Pinus ponderosa* and *Ephedra major*), the most prominent features are phenylpropanoid-associated signals of sporopollenins around 1630, 1605, 1585, 1205, 1170, 855, and 830 cm^–1^ in the Raman spectra, and around 1605, 1515, 1205, 1170, 855, 830 and 815 cm^–^ in the FTIR spectra^1^ (all vibrations are related to phenyl ring vibrations). Furthermore, some taxa, such as grasses (e.g., Figure 1 *Festuca amethystina*) and sedges, have strong carbohydrate signals around 1450-1300 (CH_2_ and CH deformations) and 1150-900 cm^–1^ (C−O−C, C−C and C−O stretching vibrations) in the Raman spectra, and around 1200-900 cm^–1^ (C−O, C−C, C−O−C, and C−OH stretches and deformations) in the FTIR spectra. Signals related to lipids (e.g., Figure 1 *Fagus sylvatica*), around 1745 (C=O stretch), 1440 and 1300 (CH_2_ deformation), and 1070 cm^–1^ (C−C stretch) in the Raman, and around 1745 (C=O stretch), 1460 (CH_2_ deformation) and 1165 cm^–1^ (C−O−C stretching in esters) in the FTIR spectra, often show large variation within related plant species. All species show prominent protein signals around 1655 (amide I), 1450 (CH_2_ deformation), and 1260 cm^–1^ (amide III) in the Raman spectra, and around 1645 (amide I), 1535 (amide II), and 1445 cm^–1^ (CH_2_ deformation) in the FTIR spectra, with taxon-specific ratio of protein-to-carbohydrate signals. In addition to these signals, a number of species (e.g., Figure 1, *Lilium bulbiferum*) show carotenoid-associated strong Raman signals around 1520 (C=C stretching), 1155 (C-C stretching), and 1005 cm^–1^ (C-CH_3_ deformation) (Schulte et al., 2009). Carotenoids are present in low concentration in pollen, and thus they cannot be detected by FTIR spectroscopy. However, resonant Raman effect enables their measurement by FT-Raman spectroscopy, which sometimes results with the complete dominance of these carotenoid signals over spectral contributions of other biochemicals, such as proteins, sporopollenins and carbohydrates (e.g., Figure 1, *Lilium bulbiferum*).

Compared to the published results of Raman measurements with 633 nm laser excitation (see Supplementary Figure S1 in Guedes et al., 2014), the FT-Raman spectra have rather simple background, which can be easily corrected with EMSC pre-processing (Supplementary Figure S2). Moreover, compared to the published results of Raman measurements with 785 nm laser excitation (Schulte et al., 2008), the FT-Raman spectra show significantly weaker resonant Raman effect. For example, the spectrum of *Aesculus hippocastanum* excited with 785 nm shows predominant carotenoid bands at 1518 and 1156 cm^–1^, while strong signals of proteins, carbohydrates and sporopollenins were recorded only after the prolonged photodestruction of the sample with 633 nm laser (see Figure 1 in Schulte et al., 2008). On the other hand, the FT-Raman spectrum of *Aesculus hippocastanum* has weak carotenoid signals and strong signals of proteins, carbohydrates and sporopollenins (Supplementary Figure S3).

### Overall Assessment of Pollen Composition

The large and extremely diverse set of measured species, covering 42 plant families, has enabled assessment of major biochemical differences and similarities between pollen species. The correlation coefficients of spectra were calculated in order to assess major patterns within and between taxa. In addition, PCA was used to estimate predominant spectral differences, and indirectly to assess principal differences in chemical composition of pollen.

**FIGURE 2:**
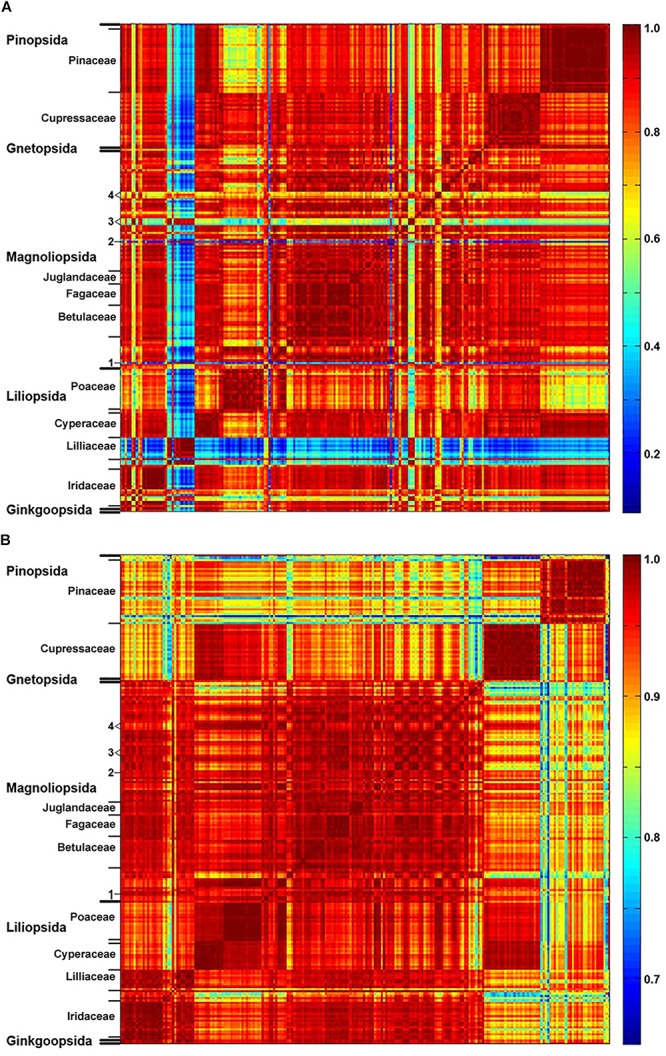
Correlation between spectroscopic data and taxonomy. Matrices of correlation coefficients calculated from: **(A)** FT-Raman and **(B)** FTIR spectra of 219 species (*Dataset I*, average spectra of 3 replicates), with depiction of plant classes and families (in addition: 1, *Taraxacum officinale*; 2, *Hibiscus trionum*; 3, *Eschscholzia californica*, *Glaucium flavum*, *Papaver lapponicum*; 4, *Ranunculus repens*, *Ranunculus acris*, *Ranunculus lanuginosus*).

The matrices of correlation coefficients (Figure 2 and Supplementary Figure S4) show that plant families have relatively uniform and specific pollen composition, specifically that pollen of related species share common chemical features. Such property is apparent, for example, for pollens of Pinaceae, Cupressaceae, Fagaceae, Betulaceae, Poaceae and Cyperaceae. However, FT-Raman spectral set has higher spectral variability than infrared set, as shown by the larger range of correlation coefficients, with a number of taxa showing specific spectral patterns (Figure 2A). For example, FT-Raman spectra of Liliaceae (Figure 2A) are extremely different compared to spectra of a majority of angiosperms (including other monocots), while the corresponding infrared spectra (Figure 2B) show considerably lower level of variability. Moreover, a number of species show specific FT-Raman spectral fingerprints, such as large variations for congeneric species of *Iris* and *Papaver* (see Iridaceae and number 3 markings in Figure 2A). In all the cases the large spectral variability within the FT-Raman data set is driven by the strong Raman signals of the carotenoids that overshadow spectral contributions from other chemicals, as illustrated previously by the spectrum of *Lilium bulbiferum* (Figure 1A).

**FIGURE 3:**
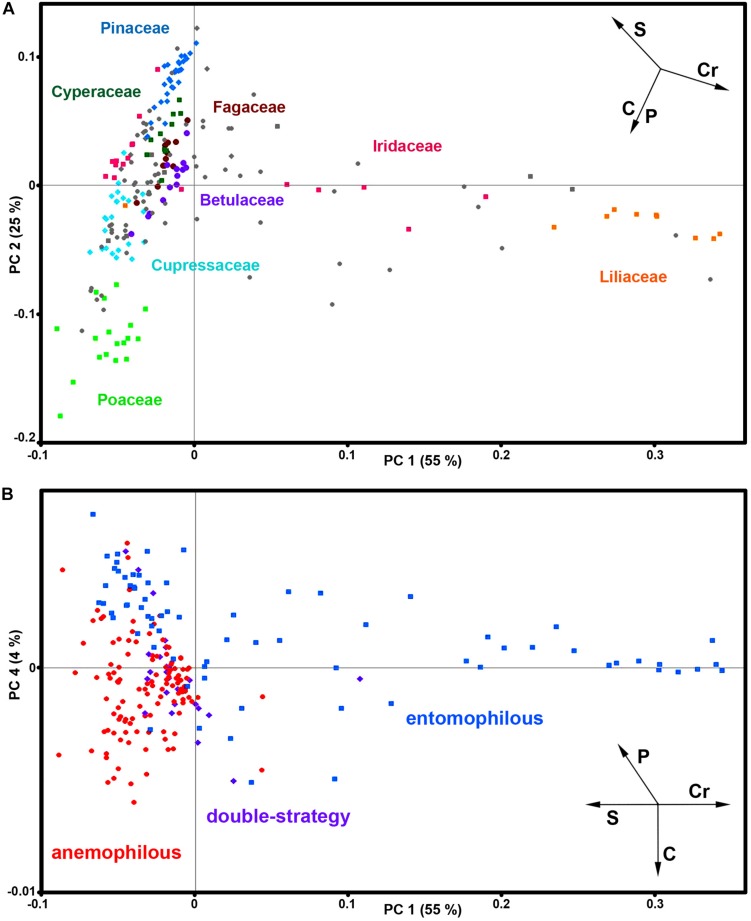
Correlation between FT-Raman data and taxonomy. PCA score plots of FT-Raman dataset containing 219 species (*Dataset I*, average spectra of 3 replicates) with: **(A)** depiction of plant classes (■ monocots; ◆ gymnosperms; ● eudicots and magnoliids) and families (Pinaceae, Cupressaceae, Betulaceae, Fagaceae, Poaceae, Cyperaceae, Liliaceae, and Iridaceae), and **(B)** depiction of pollination strategies (blue ■ entomophilous; red ● anemophilous; purple ◆ double-strategy) species. Vectors are approximating the increase in relative amount of proteins (P), sporopollenins (S), carbohydrates (C) and carotenoids (Cr). The percent variances for the first five PCs are 54.77, 25.08, 6.43, 4.41, and 2.31.

**FIGURE 4:**
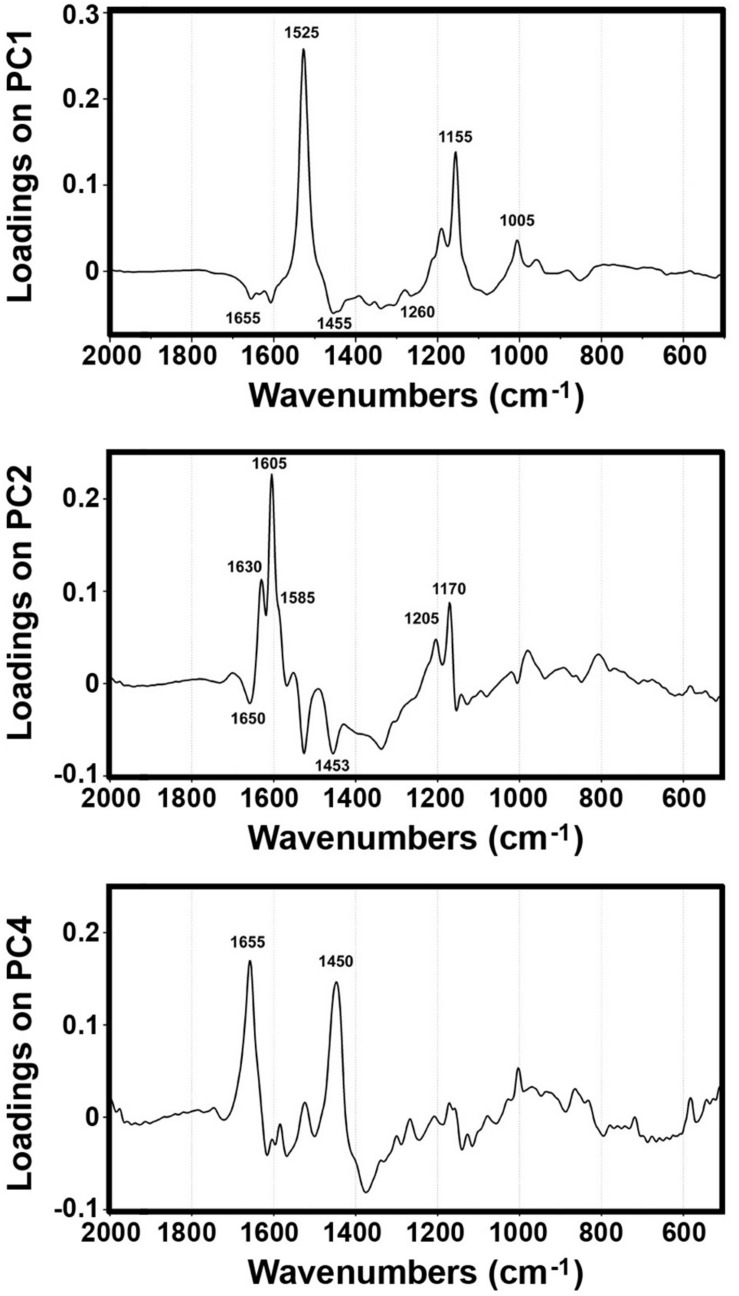
Loadings plots on the principal components 1, 2 and 4 of FT-Raman dataset containing 219 species (Dataset I, average spectra of 3 measurements).

The PCA of FT-Raman data shows that the predominant spectral differences are the result of variations of bands associated with carotenoids, sporopollenins, carbohydrates and proteins (Figures 3, 4). The PCA plots have high factor loadings associated with carotenoids (positive loadings) at 1523, 1155, and 1005 cm^–1^, and proteins (negative) at 1655, 1455, and 1260 cm^–1^ in PC 1, and sporopollenins at 1630, 1605, 1585, 1205, and 1170 cm^–1^, and proteins (negative) at 1650 and 1453 cm^–1^ in PC 2 (Figure 4). Therefore, it is evident that the predominant information from FT-Raman spectral data is on pollen grain wall chemicals. The PCA score plot in Figure 3 indicates scores for the selected plant families with relatively high number of species represented in the data set. Similar as the matrices of correlation coefficients (Figure 2 and Supplementary Figure S4), the score plot shows that the majority of Liliaceae, as well as the number of Iridaceae species, have quite different pollen chemistry (in particular, carotenoid content) compared to the rest of measured pollen species. All the major families, apart from Iridaceae, show relatively good clustering, indicating taxon-specific chemistry. For example, the separation of relatively related clades Poaceae and Cyperaceae (both Poales), as well as Pinaceae and Cupressaceae (both Pinales), is mostly driven by the difference in their sporopollenin and carbohydrate content and composition.

Furthermore, the PCA score plot of PCs 1 and 4 indicates a trend in pollen chemistry composition based on pollination mode (Figure 3). Anemophilous (wind pollinated) and entomophilous (insect pollinated) species show different tendencies based on relative content of protein, carbohydrates and carotenoids. The PCA loading plots have high factor loadings associated with carotenoids and proteins in PC 1, and proteins (positive) at 1655 and 1450 cm^–1^, and carbohydrates (negative) at 1450-1300 and 1150-1050 cm^–1^ in PC 4 (Figure 4). In general, anemophilous species have low content of carotenoids and proteins, and high content of carbohydrates, compared to entomophilous species. This is in agreement with the published studies, showing that insect foragers prefer plants with high-protein pollen content (Roulston et al., 2000), while anemophilous species produce pollen with high carbohydrate content (Speranza et al., 1997; Wang et al., 2004). The evolutionary explanation is that production of proteins has higher metabolic cost, and thus plants with non-rewarding pollen (anemophilous, self-pollinators, and nectar rewarders) have pollen with higher carbohydrate content. Although the spectroscopy results are in agreement with previous studies on pollination mode and pollen chemistry, it is possible that the trends present in Figure 3 are driven by plant relatedness, specifically that pollen of related species share common chemical features. Therefore, further studies are needed, preferably on a group of closely related species presenting different pollination modes.

**FIGURE 5:**
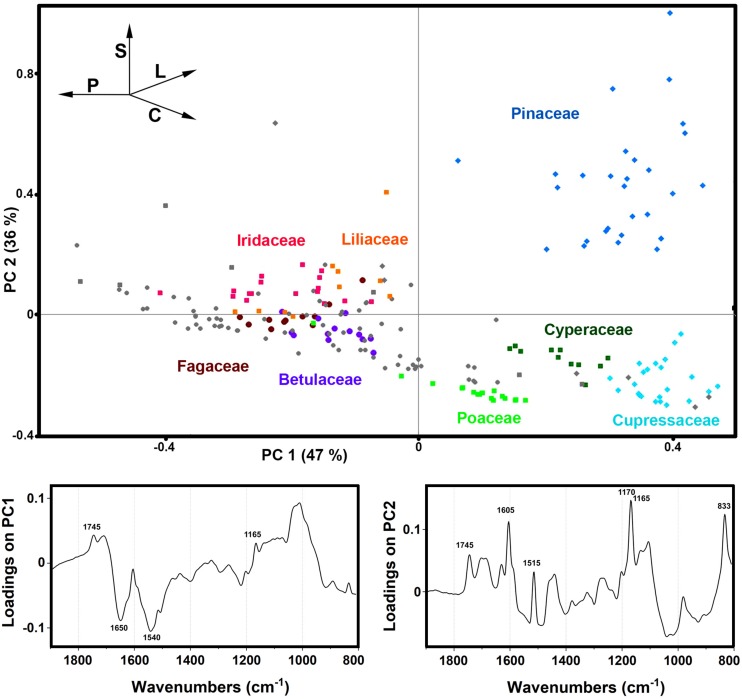
Correlation between FTIR data and taxonomy. PCA score and loading plots of FTIR dataset containing 219 species (*Dataset I*, average spectra of 3 replicates) with: depiction of plant classes (■ monocots; ◆ gymnosperms; ● eudicots and magnoliids) and families (Pinaceae, Cupressaceae, Betulaceae, Fagaceae, Poaceae, Cyperaceae, Liliaceae, and Iridaceae). Vectors are approximating the increase in relative amount of proteins (P), sporopollenins (S), carbohydrates (C), and lipids (L). The percent variances for the first five PCs are 47.44, 35.59, 4.12, 2.53, and 2.18.

The PCA of the FTIR data shows that the predominant spectral differences are the result of variations of bands associated with proteins, carbohydrates and sporopollenins (Figure 5). The PC loading plots have high factor loadings associated with proteins (negative loadings) at 1650 and 1540 cm^–1^, carbohydrates (positive) at 1050-950 cm^–1^ and lipids (positive) at 1745 and 1165 cm^–1^ in PC 1, and carbohydrates (negative) at 1050-950 cm^–1^, sporopollenins (positive) at 1605, 1515, 1170 and 833 cm^–1^, and lipids (positive) at 1745 and 1165 cm^–1^ in PC 2. The PCA score plot in Figure 5 indicates scores for the selected plant families highlighted in the PCA score plot of Raman data (Figure 3). Similar as for the Raman data, the major plant families show taxon-specific clustering. For example, analogous to the Raman data set, the separation of relatively related clades Poaceae and Cyperaceae (both Poales), as well as Pinaceae and Cupressaceae (both Pinales), is mostly driven by the difference in their sporopollenin and carbohydrate content and composition. This issue has already been mentioned in our previous studies (Zimmermann and Kohler, 2014), and it will be discussed in more details later in this paper. The main difference between the FTIR and FT-Raman data is lack of the carotenoid-driven outliers in the FTIR that were present in the Raman data (in particular, Liliaceae). Another difference is relative large variation in the FTIR data driven by the lipid content, which was mostly lacking in the FT-Raman data. The issue of carotenoid and lipid content will be tackled in more details below when we discuss strategies for quantification of relative chemical composition of pollen.

### Relative Chemical Composition of Pollen

A primary drawback of PCA of vibrational spectral data is its reduced interpretability due to complex loadings. Therefore, the data matrices containing complete set of spectra were deflated by using spectra of standard compounds (Zimmermann and Kohler, 2014). That way, estimates of chemical composition of pollen were obtained regarding principal type of compounds: triglyceride lipids, proteins, carbohydrates and carotenoids. It should be noted that this procedure is not a replacement for aforementioned PCA, particularly if residual spectral component is large and contains important variability information. However, the obtained eigenvalues can be good proxies for estimating relative chemical composition of pollen and for simple visualization of pollen composition.

**FIGURE 6:**
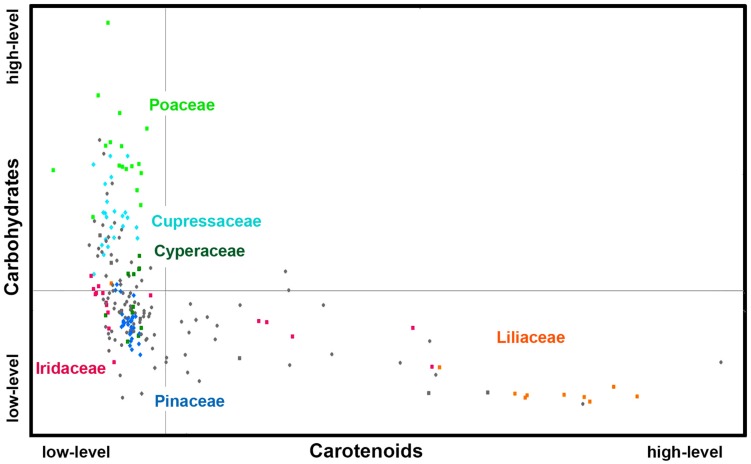
Scatter plot of eigenvalues from the reduced FT-Raman data set (*Dataset I*, average spectra of 3 replicates), obtained by modeling spectral contribution of carbohydrates and carotenoids, with depiction of plant classes (■ monocots; ◆ gymnosperms; ● eudicots and magnoliids) and families (Pinaceae, Cupressaceae, Poaceae, Cyperaceae, Liliaceae, and Iridaceae). Axis scales are in relative units and are based on deflation eigenvalues.

**FIGURE 7:**
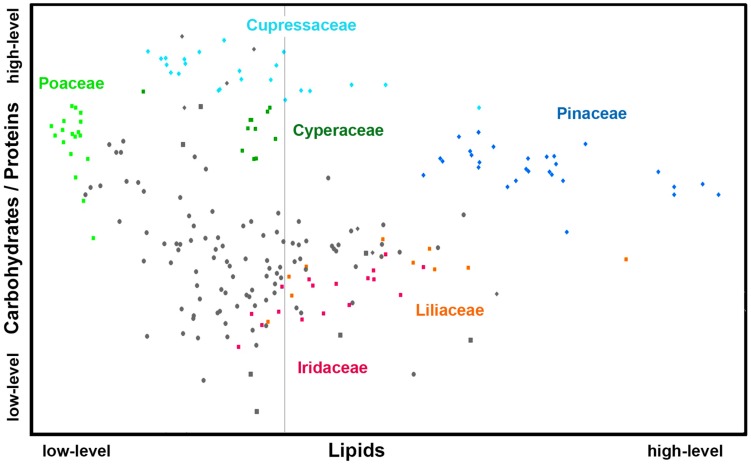
Scatter plot of eigenvalues from the reduced FTIR data set (*Dataset I*, average spectra of 3 replicates), obtained by modeling spectral contribution of carbohydrates and proteins (expressed as C/P ratio), and lipids with depiction of plant classes (■ monocots; ◆ gymnosperms; ● eudicots and magnoliids) and families (Pinaceae, Cupressaceae, Poaceae, Cyperaceae, Liliaceae, and Iridaceae). Axis scales are in relative units and are based on deflation eigenvalues.

Figures 6, 7 show that relative composition of pollen has big variations regarding carotenoids and triglycerides. These type of compounds show substantial variations even for congeneric species. For example, a number of far-related genera, such as *Quercus*, *Iris*, *Pinus*, and *Juniperus* show large variations for congeneric species regarding triglycerides, as well as *Papaver*, *Lilium* and *Iris* regarding carotenoids (Supplementary Figures S5, S6, respectively). On the other hand, relative composition regarding carbohydrates and proteins is quite taxa-specific. These two type of compounds show negative linear correlation in the FTIR dataset (Supplementary Figure S7). It has been known that the pollen protein content is very similar for congeneric species, and it can be even similar for confamiliar species (Roulston et al., 2000). Based on this and the aforementioned linear correlation between pollen carbohydrates and proteins, it can be concluded that pollen carbohydrate content is highly conserved within genera and families as well. Moreover, there is a clear trend in chemical composition for anemophilous and entomophilous species, which is consistent with FT-Raman results in Figure 3 and our previous finding (Zimmermann and Kohler, 2014). Pollen of anemophilous plants have much higher relative content of carbohydrates, defined as carbohydrate-to-protein ratio, as compared with entomophilous plants (Supplementary Figure S7).

These results are in agreement with the published studies. As mentioned previously, insect pollinated species have in general higher protein content and lower carbohydrate content than anemophilous plants (Speranza et al., 1997; Roulston et al., 2000). Similarly to our results, the study by Roulston et al. (2000), has revealed that anemophilous pollens contain significantly less protein (average value 25.8% protein dry mass content) than zoophilous (animal pollinated) pollens (average value 39.3%). However, the authors have stipulated that this discrepancy could arise from a sampling and measurement bias. They have stated that due to analytical limitations and relative ease of collecting anemophilous pollens, anemophilous species are always overrepresented in the data set. The main reason is that standard analyses of pollen protein content requires 1-1000 mg of pollen (Roulston et al., 2000), thus favoring anemophilous plants that produce large quantities of pollen. Although in our vibrational study the sampling set was relatively balanced regarding number of anemophilous and entomophilous species, the sampling bias cannot be entirely disregarded. It should be noted that vibrational microspectroscopy can measure single pollen grains (Zimmermann et al., 2016), and therefore quantification of pollen proteins by spectroscopy approach would be equally applicable to anemophilous and zoophilous pollen.

### Quantitative Measurement of Pollen Protein Content

The exploratory data analyses, as the ones presented above, are offering valuable information on relative chemical composition of pollen, as well as on chemical differences within and between taxa. However, the next important question to address is whether the vibrational data on pollen contains valuable quantitative biochemical information to allow the prediction of absolute chemical composition. Quantitative chemical analysis of complex biological samples, such as composition of biomass or biofluids, is readily obtained by combining vibrational spectroscopy with multivariate regression, such as PLSR (Zimmermann and Kohler, 2013; Kosa et al., 2017). Therefore, we have conducted PLSR analyses on FT-Raman and FTIR datasets for predicting protein mass fraction of pollen (percentage of protein by dry mass). PLSR models were validated, using the full cross validation method, against protein mass fraction values for 35 species obtained from Roulston et al. (2000). The analyzed species include all major taxa of seed plants (see Supplementary Table S2), and had extensive range of protein content, from 8.8 to 43.1% of protein content by dry mass.

The *R*^2^ values for the PLSR models were 0.53 and 0.49 for FT-Raman and FTIR models respectively, with RMSE errors of approx. 15% (Table 2). PLSR regression coefficients are summarizing the relationship between spectral variables and protein mass fraction values. As can be seen, the spectral features associated with proteins are present in the regression coefficients at 1640-65 cm^–1^ (amide I), 1452 cm^–1^ (CH_2_ deformation) and 1006 cm^–1^ (phenylalanine sidechain vibrations) for FT-Raman dataset, and at 1630-1670 cm^–1^ (amide I) and 1515-1560 cm^–1^ (amide II) for FTIR dataset (Supplementary Figure S8). FT-Raman model was based on a larger number of components (*A*_*op*__*t*_ = 12) than the model based on FTIR data (*A*_*op*__*t*_ = 6). This is probably due to relatively strong protein-related signals in FTIR spectra, compared to FT-Raman spectra where protein signals are often overlapped by stronger signals associated with sporopollenins and carotenoids. It should be noted that the reference data for the PLSR models was based on literature values, and not on an actual measurements of studied samples. It can be assumed that prediction models will improve when actual protein reference values for measured samples are used, and when they are restricted to phylogenetically related taxa, for example plant orders and families. Moreover, there is a great potential of vibrational spectroscopy for direct measurement of not only protein content of pollen, but other constituents, such as carbohydrates and carotenoids, as well.

**TABLE 2:** PLS regression results between vibrational spectra and protein content for 35 pollen species (*N* = 35).

PLSR parameter	FTIR	FT-Raman
RMSE	6.45%_*wt*_ (15%)	6.19%_*wt*_ (14%)
*R*^2^	0.487	0.527
R	0.698	0.726
*A*_*opt*_	6	12

*%_*wt*_ = protein mass fraction (% of protein by dry mass).*

### Chemical Composition of Phenylpropanoids in Pollen Grain Wall

Pollen wall is extremely resilient structure, both physically and chemically, protecting generative cells from environmental stress, including ultraviolet light, temperature, excessive water loss and gain, and microbial damage. In general, pollen wall is comprised of two layers with distinct chemical composition: exine, an outer layer, and intine, an inner layer (Blackmore et al., 2007). Exine is the most complex and resilient plant extracellular matrix, and is predominantly composed of sporopollenins, an extremely robust, chemically resistant and complex biopolymers (Jiang et al., 2013). Sporopollenins are a group of chemically related polymers composed of covalently coupled derivatives of fatty acid and aromatic phenylpropanoid building blocks, with significant taxon-specific variations in chemical composition (Dominguez et al., 1999; Blackmore et al., 2007; Jiang et al., 2013; Li et al., 2019). Production of phenylpropanoids in plants is induced by solar ultraviolet radiation (UV-B) via the phenylpropanoid pathway, the same pathway responsible for synthesis of similar complex biopolymers, such as lignin and suberin. Unlike the intine and the grain interior (i.e., vegetative and generative cells), which are synthesized under the control of the gametophytic genome, sporopollenins are synthesized in the tapetum under the control of the sporophytic genome (Piffanelli et al., 1998; Blackmore et al., 2007). Therefore, sporopollenin measurements reveal important information on parent plants (sporophytes), in particular concerning plant-environment interactions.

A number of studies have shown response of sporopollenin chemistry to variation in UV-B radiation levels received by sporophytes for a range of different plant species, such as conifers, grasses and legumes (Rozema et al., 2001, 2009; Willis et al., 2011; Lomax et al., 2012; Jardine et al., 2016; Bell et al., 2018). Recent studies have shown that the FTIR analysis of UV-B-absorbing phenylpropanoids in sporopollenins of pollen and spores could provide very valuable record of solar-UV radiation received by plants, which is of high interest in palaeoclimatic and palaeoecological fields. The primary phenylpropanoids in pollen, such as derivatives of *p*-coumaric, ferulic and sinapic acids, have specific vibrational bands in both infrared and FT-Raman spectra, and thus specific spectral regions can be selected and analyzed in detail in order to obtain characteristic chemical fingerprints of pollen cell wall (Bagcioglu et al., 2015). The main spectral regions (i.e., aromatic regions) for characterization of phenylpropanoids are 860-800 cm^–1^ in infrared spectra, associated with phenyl C-H out-of-plane deformations, and 1650-1580 cm^–1^ in FT-Raman spectra, associated with phenyl C=C stretching vibrations.

**FIGURE 8:**
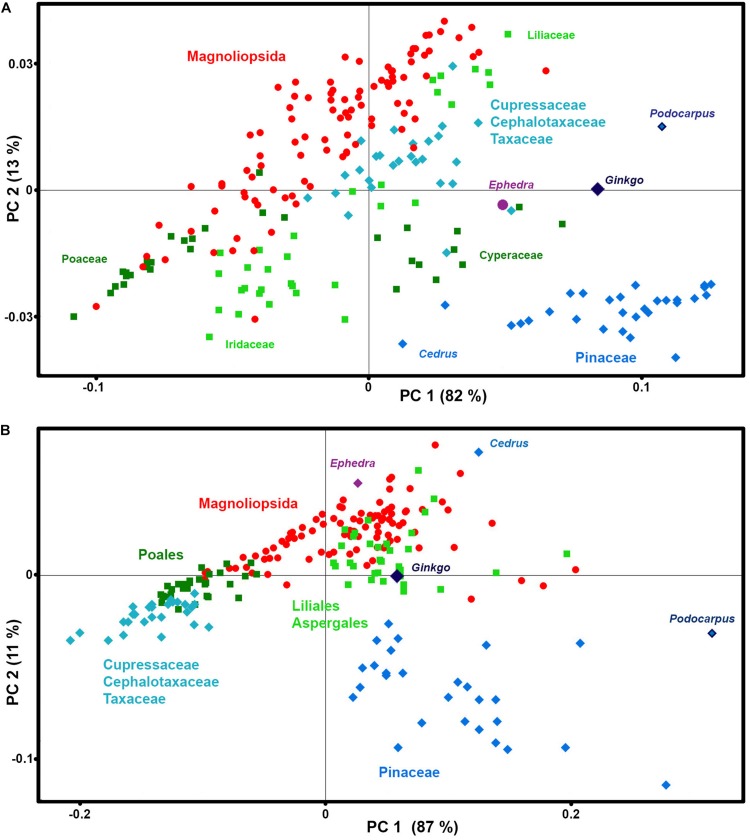
Correlation between phenylpropanoids-related spectral data and taxonomy. PCA score plots of: **(A)** FT-Raman and **(B)** FTIR datasets, containing 219 species (*Datasets II*) with depiction of plant classes (■ monocots; ◆ gymnosperms; ● eudicots and magnoliids), orders, families and selected species.

The PCA data analysis of these spectral regions shows that the majority of taxa have phylogeny-based similarities in chemical composition of phenylpropanoids (Figure 8). In accordance with our previous finding (Bagcioglu et al., 2015), *Cedrus* is a noteworthy outliers, showing quite different chemistry when compared to the rest of Pinaceae species, with a higher ratio of ferulic-to-*p*-coumaric acid derivatives in sporopollenin compared to the other species. In general, gymnosperms show much higher chemical variability of phenylpropanoids than angiosperms, with substantial differences between Cupressaceae, Cephalotaxaceae, Pinaceae, *Podocarpus*, *Ginkgo*, and *Ephedra* (For example, see differences in 900–800 cm^–1^ region for FTIR spectra of *Pinus ponderosa* and *Ephedra major* in Figure 1B). This is not surprising, since all major families of gymnosperms have diverged in Permian-Triassic periods (300-200 Ma) (Lu et al., 2014), much earlier that angiosperm families.

In addition, the analysis has revealed a difference in chemical composition of phenylpropanoids between sedges (Cyperaceae) and grasses (Poaceae). While in both cases the predominant signals belong to *p*-coumaric acid at 830 cm^–1^ in FTIR and 1605 cm^–1^ in FT-Raman, grasses have additional signals associated with ferulic acid at 850 and 1605 cm^–1^ in FTIR and FT-Raman respectively, while sedges have signals associated with sinapic acid at 815 and 1595 cm^–1^ in FTIR and FT-Raman respectively. This is in accordance with phenylpropanoid studies of plant vegetative tissues which have shown that grass cell walls are characterized by ferulic and *p*-coumaric acids, while sedges contain sinapic and *p*-coumaric acids (Bogucka-Kocka et al., 2011; De Oliveira et al., 2015). Sinapic acid is rarely detected in plant tissues, and it has been hypothesized that its presence in tissues of a number of *Carex* species can be associated with the humidity of plants’ habitats (Bogucka-Kocka et al., 2011).

### Comparative Assessment of FT-Raman and FTIR: A Case Study on Monocots and *Iris*

**FIGURE 9:**
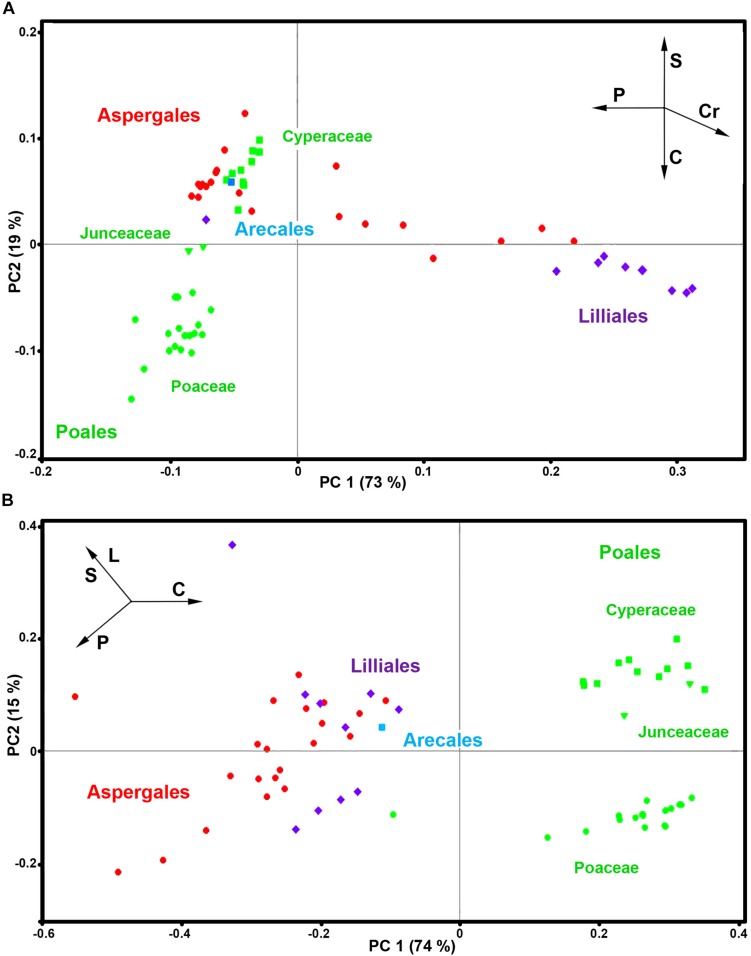
PCA score plots for monocots (Liliopsida): **(A)** FT-Raman and **(B)** FTIR datasets, containing 63 species (*Datasets I*, average spectra of 3 replicates) with depiction of plant order (green - Poales; red - Asperagales; purple – Liliales; blue - Arecales) and families (■ Cyperaceae; Juncaceae; ● Poaceae). Vectors are approximating the increase in relative amount of proteins (P), sporopollenins (S), carbohydrates (C), lipids (L), and carotenoids (Cr). The percent variances for the first five PCs are 73.09, 18.67, 3.20, 1.78, and 1.39 for FT-Raman, and 73.92, 14.82, 3.88, 2.66, and 1.46 for FTIR.

Here we will demonstrate the benefits of pollen phenotyping by both FT-Raman and FTIR methods by taking a more detailed look on spectral data of monocots (Monocotyledons, Liliopsida). Monocots are large clade, covering a variety of habitats, and include quite diverse group of plants such as lilies, agaves and sedges, as well as grasses which are economically the most important group of plants. The PCA analyses of FT-Raman and FTIR data reveal corresponding and complementary information on pollen chemistry (Figure 9 and Supplementary Figures S4, S9).

The PCA plots have high factor loadings associated with carotenoids (positive loadings) at 1526, 1156 and 1007 cm^–1^, and proteins (negative) at 1657 and 1450 cm^–1^ in PC 1, and sporopollenins (positive loadings) at 1630, 1603, 1204 and 1171 cm^–1^, and carbohydrates (negative) at 1460-1300 and 1150-1000 cm^–1^ and carotenoids (negative) at 1526, and 1156 cm^–1^ in PC 2 (Supplementary Figure S9a). In contrast, FTIR data shows large variation in carbohydrate-to-protein and carbohydrate-to-lipid ratios. The PC loading plots have high factor loadings associated with proteins (negative loadings) at 1640 and 1530 cm^–1^, carbohydrates (positive) at 1050-950 cm^–1^, lipids (negative) at 1744 and 1165 cm^–1^, and sporopollenins (negative) at 1605, 1514, 1167, and 831 cm^–1^ in PC 1, and sporopollenins (positive) at 1603, 1512, 1165, and 831 cm^–1^, lipids (positive) at 1744 and 1165 cm^–1^, and proteins (negative) at 1645 and 1530 cm^–1^ in PC 2 (Supplementary Figure S9b).

Both FT-Raman and FTIR can easily distinguish between the two, predominantly anemophilous, families: grasses (Poaceae) and sedges (Cyperaceae). However, this discrimination is based on relative protein-to-lipid ration in FTIR data, which is high in grasses and low in sedges, while in FT-Raman data it is based on carbohydrate-to-sporopollenin ratio, which is high in grasses and low in sedges. Regarding the three, predominantly entomophilous, families (Xanthorrhoeaceae, Liliaceae and Iridaceae) it is evident that information obtained by FT-Raman has no equivalence in FTIR. The predominant spectral variability in FT-Raman data belongs to relative amount of carotenoids, and, to a less extent, to sporopollenins as well. These results are in accordance with previous studies that have shown significant reserves of starch nutrients in Poaceae and Cyperaceae pollen, while pollen grains of Iridaceae, Xanthorrhoeaceae, Liliaceae, and Arecaceae is predominantly starchless (Franchi et al., 1996).

The large dataset has enabled us to study differences for congeneric species, in particular *Iris* genus comprising spectral data on 17 species. *Iris* is the largest genus of Iridaceae, comprising approx. 250 entomophilous species that inhabit the Earth’s North Temperate Zone (Mathew, 1981). *Iris* flowers have elaborate and versatile pollinator attractants, including different color patterns of tepals and sepals with specific orientation and distinctive nectar guides, floral odors, nutritive nectars and pollen, as well as non-nutritive forms of reward, such as shelter and thermal energy (Sapir et al., 2006; Vereecken et al., 2013; Imbert et al., 2014; Guo, 2015; Pellegrino, 2015). Regarding pollen chemistry, triglycerides are the primary nutrient reserve in *Iris* pollen since the grains are starchless (Franchi et al., 1996).

**FIGURE 10:**
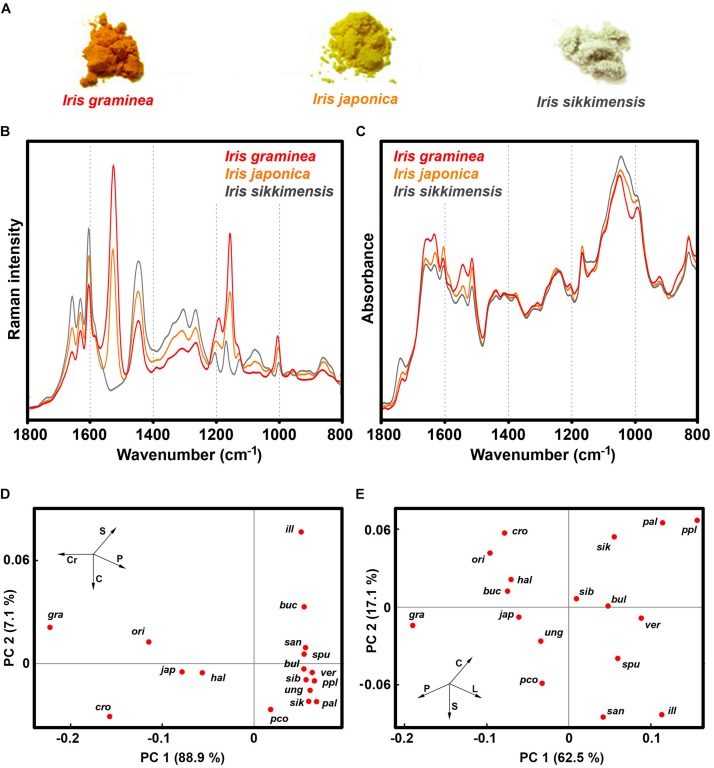
Vibrational spectroscopy of *Iris* pollen: **(A)** Bulk samples of pollen for *I. graminea*, *I. japonica* and *I. sikkimensis*; **(B)** FT-Raman, and **(C)** FTIR spectra of the aforementioned pollen samples; PCA score plots for **(D)** FT-Raman, and **(E)** FTIR spectral data of pollen comprising 17 *Iris* species: *ill - I. illyrica, ppl - I. pseudopallida, pal - I. pallida, sik - I. sikkimensis, ver - I. versicolor, pco - I. pseudacorus, san - I. sanguinea, sib - I. sibirica, bul - I. bulleyana, gra - I. graminea; spu - I. spuria, cro - I. crocea, ori - I. orientalis, hal - I. halophila, ung - I. unguicularis, jap - I. japonica, buc - I. bucharica*.

Our study shows that *Iris* species have a large variation of pollen chemistry (Figure 10 and Supplementary Figure S4). The species with large content of carotenoids, such as *I. graminea*, *I. orientalis*, *I. japonica* and *I. crocea*, have relatively low content of lipids and carbohydrates, and high content of proteins (Figure 10 and Supplementary Figure S10). It should be noted that our previous FTIR study has shown that pollen lipids between various species of *Iris* can vary tenfold, as for example between *I. pallida* and *I. graminea* (Zimmermann and Kohler, 2014). This could indicate differences in germination and pollen tube growth, considering the roles of triglycerides in other plants (Rodriguez-Garcia et al., 2003). Pollen with high amount of lipid and carbohydrate nutrients is colorless, such as *I. sikkimensis*, *I. pallida*, *I. unguicularis*, and *I. spuria* (Figure 10 and Supplementary Table S3). The spectral results show no clear clustering with phylogeny of Iris genus. For example, *I. unguicularis* is considered to belong to clade Siphonostylis, a sister group (and proposed separate genus) to the rest of *Iris* taxa (Mavrodiev et al., 2014). However, in PCA scores plots for both FT-Raman and FTIR spectral data, *I. unguicularis* PC1 and PC2 scores are close to the median values (Figure 10). Limniris clade (Mavrodiev et al., 2014), comprising *I. versicolor*, *I. pseudacorus*, *I. sanguinea*, *I. sibirica*, and *I. bulleyana*, shows relatively good clustering, though several far-related taxa, such as *I. unguicularis* and *I. spuria*, have similar score values (Figure 10). In contrast, Chamaeiris clade (Mavrodiev et al., 2014), comprising *I. graminea*, *I. spuria*, and *I. orientalis*, shows large difference of score values for both FTIR and FT-Raman data (Figure 10). These results indicate that chemical phenotype of *Iris* pollen, as measured by vibrational spectroscopy, is somewhat unrelated to the genotype. In general, the results clearly show a large variability in pollen chemistry for congeneric species, in particular regarding content of proteins, lipids and carotenoids. In fact, the study indicates large variations even for subgenus clades, such as Chamaeiris clade (Mavrodiev et al., 2014). These extensive differences in pollen biochemistry indicate that congeneric species can employ different reproductive strategies.

Pollen pigments, in the form of carotenoids and flavonoids, are predominantly accumulated in pollenkitt, a sticky lipid-rich pollen coat that covers exine and which is developed under control of sporophytic genome (Pacini and Hesse, 2005; Fambrini et al., 2010). The function of pollen pigments has not been sufficiently studied, but they probably have several functional roles, such as light screening and oxidative stress defense (Stanley and Linskens, 1974; Fambrini et al., 2010). Our study indicates that, in addition to aforementioned functions, pollen carotenoids can also have role in plant signaling, serving as attractor for pollinators to indicate protein-rich pollen. Conversely, pollen coloration could have protective role as nectar guides to direct pollinators toward nectar reward. This notion is partly supported by the fact that brightly colored pollen predominantly belongs to nectar-bearing *Iris* species, such as those belonging to *Iris* subg. *Limniris* series *Spuriae* (see Supplementary Table S3), i.e., Chamaeiris clade (Mavrodiev et al., 2014). In those species, primarily floral reward is most likely nectar and not pollen. It has been observed that bumblebees form expectations, based on flower color, on what type of reward a plant will offer (Nicholls and De Ibarra, 2014; Muth et al., 2015). For example, bumblebees can simultaneously learn floral cues associated with pollen and nectar rewards (Muth et al., 2015). However, it is possible that pollen color is the indirect target of selection through genetic associations with some other trait under selection, such as petal color.

## Conclusion

Vibrational spectroscopies, coupled with multivariate data analysis, have shown great potential for simple and economical chemical characterization, identification and classification of pollen. This study has demonstrated that high-quality Raman spectra of pollen, comprising all principal taxa of seed plants, can be obtained by FT-Raman spectroscopy. All FT-Raman spectra were devoid of strong fluorescence background, have high signal-to-noise ratio, and contain clear signals of not only pollen intracellular constituents (lipids, carbohydrates and proteins), but grain wall constituents as well (pigments and sporopollenins). Thus, FT-Raman spectra are superior than corresponding spectra obtained by dispersive Raman spectrometers. In combination with FTIR spectroscopy, FT-Raman spectroscopy is obtaining comprehensive information on pollen biochemistry. Specifically, FT-Raman spectra are strongly biased toward chemical composition of pollen wall constituents, namely sporopollenins (namely phenylpropanoids) and carotenoids, while FTIR spectra are over-representing chemical constituents of the grain interior, such as lipids and carbohydrates. Since chemical composition of pollen depends both on sporophytic genome, which controls expression of sporopollenins and carotenoids, and on gametophytic genome, which controls expression of intracellular lipid and carbohydrate nutrients, it means that each technique can provide unique information on certain aspects of plant genome and pollen development.

The study has demonstrated that the main biochemical constituents of pollen can be identified, and that relative chemical content of pollen can be estimated. Moreover, absolute values of protein content, and probably other chemicals as well, can be obtained by multivariate regression. Our results show a large variability in pollen chemistry for families, genera and even congeneric species, revealing wide range of reproductive strategies. The information on pollen’s chemical patterns for major plant taxa should be of value for various studies in plant biology and ecology, including aerobiology, community ecology, palaeoecology, plant-pollinator interactions, and climate effects on plants.

## Data Availability Statement

All measured FTIR and FT-Raman spectral data is available in the Supplementary Material.

## Author Contributions

BZ conceived the research idea, contributed to the pollen sampling, performed the FTIR measurements, analyzed the data, and wrote the manuscript. AK and BZ conceived and designed the experiments, discussed and revised the manuscript. AK performed the FT-Raman measurements.

## Conflict of Interest

The authors declare that the research was conducted in the absence of any commercial or financial relationships that could be construed as a potential conflict of interest.
