# Supplementary material for: Chemical Analysis of Pollen by FT-Raman and FTIR Spectroscopies
Source: Front Plant Sci. 2020 Mar 31;11:352. doi: 10.3389/fpls.2020.00352 (PMC7136416; doi:10.3389/fpls.2020.00352)
Supplement: Supplementary file 1 [file Data_Sheet_1.PDF]

Supplementary Material

Chemical analysis of pollen by FT-Raman and FTIR spectroscopies

A. Kenđel[1], B. Zimmermann[2,3]*

[1] Division of Analytical Chemistry, Department of Chemistry, Faculty of Science, University of Zagreb, 10000 Zagreb, Croatia
[2] Faculty of Science and Technology, Norwegian University of Life Sciences, PO Box 5003, NO1432 Ås, Norway
[3] Division of Organic Chemistry and Biochemistry, Ruđer Bošković Institute, Bijenička 54, HR-10001 Zagreb, Croatia

*Corresponding author:
Boris Zimmermann
Department of Mathematical Sciences and Technology
Faculty of Environmental Science and Technology
Norwegian University of Life Sciences
Drøbakveien 31, 1432 Ås, Norway.
Tel: +47 6723 1576
Fax: +47 6496 5001
E-mail: boris.zimmermann@nmbu.no

**Table of Contents**	**Page**
Figure S1. Overview of analyzed pollen taxa	S-2
Table S1. List of analyzed pollen taxa	S-3
Figure S2. FT-Raman spectra of *C. betulus*, *C. humilis* and *P. lanceolata* pollen	S-9
Figure S3. FT-Raman spectra of *A. hippocastanum* pollen	S-10
Figure S4. Matrices of correlation coefficients for Liliopsida pollen	S-11
Figure S5. Scatter plot of eigenvalues from the reduced FT-Raman dataset	S-12
Figure S6. Scatter plot of eigenvalues from the reduced FTIR dataset	S-12
Figure S7. Scatter plot of eigenvalues from the reduced FTIR dataset	S-13
Table S2. List of analyzed taxa for prediction of pollen protein content	S-14
Figure S8. PLS regression coefficients	S-15
Figure S9. PCA analyses of datasets for monocots	S-16
Figure S10. PCA analyses of datasets for *Iris*	S-17
Table S3. List of analyzed *Iris* taxa	S-18

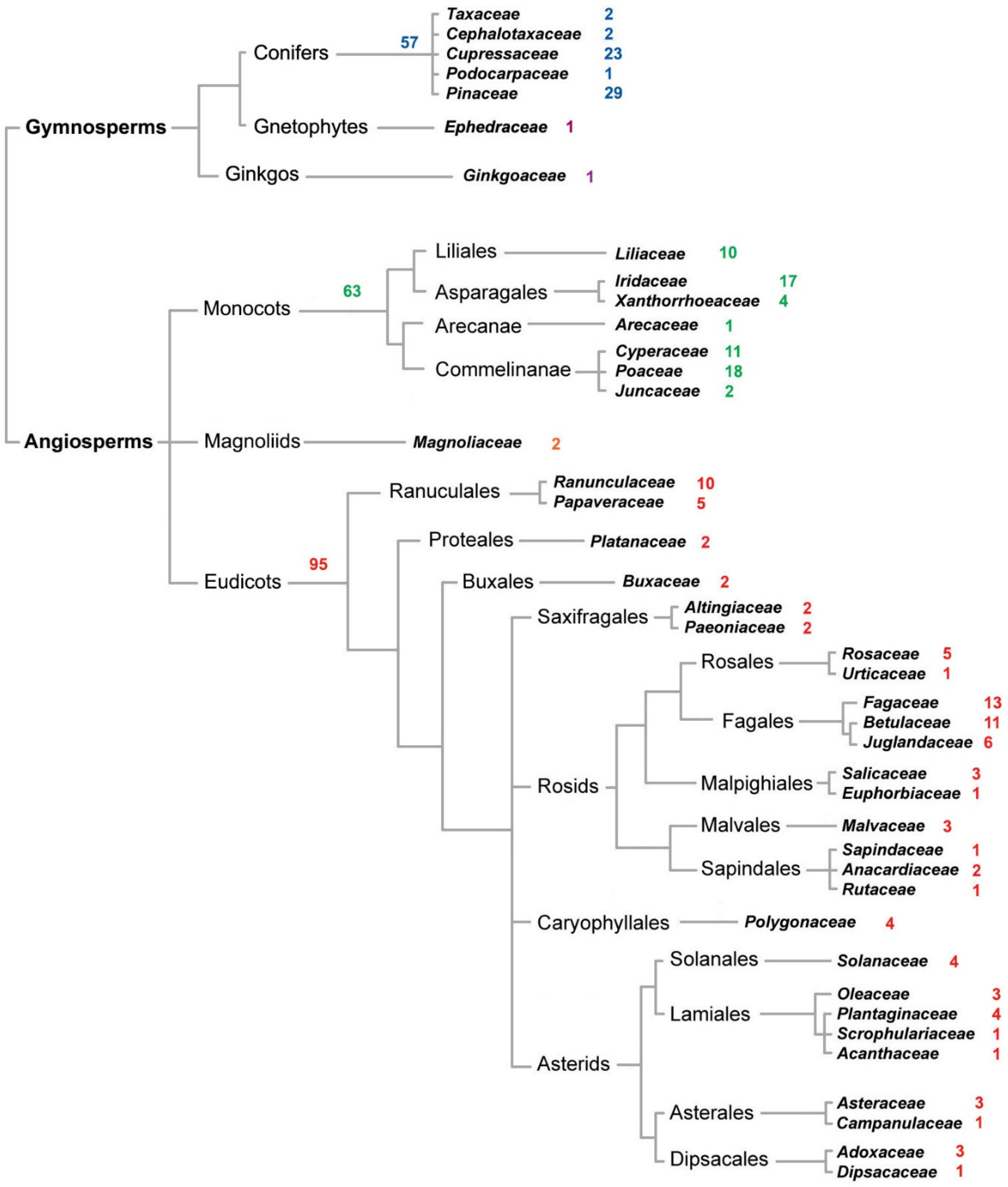

Figure S1. Tree of life of pollen species covered by the study (numbers denote number of species per taxon).

Table S1. List of analyzed pollen taxa (part I): eudicots. All samples collected in 2011. All samples collected at the Botanical Garden of the Faculty of Science, except (#) collected at the Botanical Garden of the Faculty of Pharmacy and Biochemistry.

ORDER	FAMILY	GENUS	SPECIES	COMMON NAME
Fagales	Betulaceae	*Corylus*	*C. maxima*	Filbert
			C. avellana	Common Hazel
			C. colurna	Turkish Hazel
			C. chinensis	Chinese Hazel
			C. sieboldiana	Asian Beaked Hazel
		Alnus	*A. incana*	Grey Alder
			A. glutinosa	Black Alder
		Betula	*B. ermanii*	Erman's Birch
			B. pendula	Silver Birch
			B. papyrifera	Paper Birch
		Carpinus	*C. betulus*	European Hornbeam
			C. orientalis	Oriental Hornbeam
		Ostrya	*O. carpinifolia*	European Hop-hornbeam
	Fagaceae	*Fagus*	*F. sylvatica*	European Beech
		Quercus	*Q. robur*	Pedunculate Oak
			Q. petreae	Sessile Oak
			Q. coccinea	Scarlet Oak
			Q. rubra	Northern Red Oak
			Q. cerris	Turkey Oak
			Q. faginea	Portuguese Oak
			Q. libani	Lebanon Oak
			Q. shumardii	Shumard's Oak
			Q. frainetto	Hungarian Oak
			Q. ilex	Holly Oak
	Juglandaceae	*Juglans*	*J. regia*	Persian Walnut
			J. mandshurica	Manchurian Walnut
			J. nigra	Eastern Black Walnut
		Pterocarya	*P. stenoptera*	Chinese Wingnut
			P. fraxinifolia	Caucasian Wingnut
		Carya	*C. illinoinensis*	Pecan Hickory
Rosales	Rosaceae	*Prunus*	*P. laurocerasus*	Cherry Laurel#
		Rosa	*R. canina*	Dog Rose#
			R. dumetorum	Corymb Rose#
		Sanguisorba	*S. minor*	Salad Burnet
		Filipendula	*F. vulgaris*	Fern-leaf Dropwort
	Urticaceae	*Urtica*	*U. pilulifera*	Roman Nettle#
Ranunculales	Ranunculaceae	*Aquilegia*	*A. nigricans*	Bulgarian Columbine
			A. vulgaris	European Columbine
			A. chrysantha	Golden Columbine
		Anemone	*A. coronaria*	Poppy Anemone
			A. sylvestris	Snowdrop Windflower
		Ranunculus	*R. repens*	Creeping Buttercup
			R. acris	Meadow Buttercup
			R. lanuginosus	Woolly Buttercup
		Helleborus	*H. odorus*	Fragrant Hellebore#
		Nigella	*N. damascena*	Love-in-a-Mist
	Papaveraceae	*Papaver*	*P. lapponicum*	Lapland Poppy
			P. orientale	Oriental Poppy
			P. nudicaule	Iceland Poppy
		Glaucium	*G. flavum*	Yellow Hornpoppy
		Eschscholzia	*E. californica*	California Poppy

Table S1. List of analyzed pollen taxa (part II): eudicots and magnoliids. All samples collected in 2011, except (*) collected in 2012. All samples collected at the Botanical Garden of the Faculty of Science, except (#) collected at the Botanical Garden of the Faculty of Pharmacy and Biochemistry.

ORDER	FAMILY	GENUS	SPECIES	COMMON NAME
Malpighiales	Salicaceae	*Salix*	*S. babylonica*	Peking Willow
			S. alba	White Willow
		Populus	*P. nigra*	Black Poplar*
	Euphorbiaceae	*Ricinus*	*R. communis*	Castor Bean#
Lamiales	Oleaceae	*Fraxinus*	*F. excelsior*	European Ash
			F. ornus	Manna Ash
			F. chinensis	Chinese Ash
	Plantaginaceae	*Plantago*	*P. lanceolata*	Ribwort Plantain
			P. media	Hoary Plantain
			P. major	Greater Plantain
		Digitalis	*D. purpurea*	Common Foxglove
	Scrophulariaceae	*Paulownia*	*P. tomentosa*	Empress Tree
	Acanthaceae	*Acanthus*	*A. balcanicus*	Balcan Bear's Breeches
Proteales	Platanaceae	*Platanus*	*P. x hispanica*	Hybrid (London) Plane
			P. orientalis	Oriental Plane
Saxifragales	Altingiaceae	*Liquidambar*	*L. orientalis*	Oriental Sweetgum*
			L. styraciflua	American Sweetgum
	Paeoniaceae	*Paeonia*	*P. mascula*	Wild Peony
			P. officinalis	European Peony
Sapindales	Sapindaceae	*Aesculus*	*A. hippocastanum*	Common Horse Chestnut
	Anacardiaceae	*Pistacia*	*P. lentiscus*	Mastic Tree
			P. terebinthus	Terebinth
	Rutaceae	*Dictamnus*	*D. albus*	White Dittany
Dipsacales	Adoxaceae	*Sambucus*	*S. nigra*	Black Elder#
		Viburnum	*V. rhytidophyllum*	Leatherleaf Viburnum
			V. lantana	Wayfaring Tree
	Dipsacaceae	*Cephalaria*	*C. gigantea*	Tatarian Cephalaria
Buxales	Buxaceae	*Buxus*	*B. sempervirens*	Common Box*
			B. microphylla	Japanese Box
Magnoliales	Magnoliaceae	*Magnolia*	*M. x sonlangiana*	Saucer Magnolia*
		Liriodendron	*L. tulipifera*	American Tulip Tree
Malvales	Malvaceae	*Malva*	*M. thuringiaca*	Rosy Tree Mallow
		Hibiscus	*H. trionum*	Bladder Hibiscus
		Althaea	*A. officinalis*	Common Marshmallow
Asterales	Asteraceae	*Taraxacum*	*T. officinale*	Common Dandelion
		Artemisia	*A. absinthium*	Absinthe Wormwood#
			A. vulgaris	Common Wormwood#
	Campanulaceae	*Platycodon*	*P. grandiflorus*	Chinese Bellflower
Caryophyllales	Polygonaceae	*Rumex*	*R. acetosa*	Common Sorrel#
			R. patientia	Patience Dock#
			R. aquaticus	Western Dock#
			R. alpinus	Alpine Dock#
Solanales	Solanaceae	*Nicotiana*	*N. alata*	Winged Tobacco
			N. tabacum	Common Tobacco
		Datura	*D. inoxia*	Downy Thorn-apple
		Brugmansia	*B. suaveolens*	White Angel's Trumpet

Table S1. List of analyzed pollen taxa (part III): monocots. All samples collected in 2011. All samples collected at the Botanical Garden of the Faculty of Science.

ORDER	FAMILY	GENUS	SPECIES	COMMON NAME
Poales	Cyperaceae	*Carex*	*C. pendula*	Pendulous Sedge
			C. muskingumensis	Muskingum Sedge
			C. flacca	Blue Sedge
			C. divisa	Divided Sedge
			C. ferruginea	Rusty Sedge
			C. morrowii	Variegata Sedge
			C. ornithopoda	Birdsfoot Sedge
			C. grayi	Gray's Sedge
			C. sylvatica	Forest Sedge
		Holoschoenus	*H. romanus*	Round-Headed Club-Rush
		Cladium	*C. mariscus*	Sawtooth Sedge
	Poaceae	*Secale*	*S. cereale*	Rye
		Zea	*Z. mays*	Maize
		Brachypodium	*B. retusum*	Mediterranean False-brome
			B. pinnatum	Heath False-brome
		Festuca	*F. tenuifolia*	Fine-Leaved Sheep's Fescue
			F. ovina	Sheep Fescue
			F. pratensis	Meadow Fescue
			F. drymeja	Mountain Fescue
			F. amethystina	Tufted Fescue
			F. arvernensis	Field Fescue
			F. heterophylla	Various-leaved Fescue
		Poa	*P. badensis*	Baden's Meadow-Grass
			P. pratensis	Smooth Meadow-Grass
			P. nemoralis	Wood Meadow-Grass
		Sesleria	*S. tenuifolia*	Thin-leaved Moor Grass
		Dactylis	*D. glomerata*	Orchard Grass
		Bromus	*B. erectus*	Erect Brome
		Holcus	*H. lanatus*	Common Velvet Grass
	Juncaceae	*Luzula*	*L. luzuloides*	White Wood-Rush
			L. sylvatica	Great Wood-Rush

Table S1. List of analyzed pollen taxa (part IV): monocots. All samples collected in 2011 except (*) collected in 2012. All samples collected at the Botanical Garden of the Faculty of Science.

ORDER	FAMILY	GENUS	SPECIES	COMMON NAME
Asparagales	Iridaceae	*Iris*	*I. illyrica*	Illyrian Iris
			I. pseudacorus	Yellow Iris
			I. japonica	Japanese Iris
			I. sikkimensis	Sikkim Iris
			I. versicolor	Larger Blue Flag
			I. pseudopallida	South Adriatic Iris
			I. sibirica	Siberian Iris
			I. sanguinea	Blood Iris
			I. graminea	Grass-Leaved Iris
			I. spuria	Blue Iris
			I. crocea	Crosier Iris
			I. unguicularis	Algerian Iris
			I. aphylla	Stool Iris
			I. bulleyana	Bulley's Iris
			I. pallida	Dalmatian Iris
			I. bucharica	Corn Leaf Iris
			I. orientalis	Yellow-banded Iris
	Xanthorrhoeacea	*Hemerocallis*	*H. lilioasphodelus*	Yellow Daylily
			H. citrina	Citron Daylily
		Aloe	*A. grandidentata*	Dwarf Soap Aloe
			A. striata	Coral Aloe
Liliales	Liliaceae	*Fritillaria*	*F. imperialis*	Crown Imperial*
		Lilium	*L. bulbiferum*	Orange Lily
			L. carniolicum	Carniolan Lily
			L. pumilum	Siberian Lily
			L. martagon	Martagon Lily
			L. regale	Regal Lily
			L. monadelphum	Caucasian Lily
			L. candidum	Madonna Lily
			L. japonicum	Bamboo Lily
			L. henryi	Tiger Lily
Arecales	Arecaceae	*Chamaerops*	*C. humilis*	Mediterranean Fan Palm

Table S1. List of analyzed pollen taxa (part V): gymnosperms. All samples collected in 2011 except (*) collected in 2012. All samples collected at the Botanical Garden of the Faculty of Science, except ($^{\#}$) collected at the Botanical Garden of the Faculty of Pharmacy and Biochemistry.

ORDER	FAMILY	GENUS	SPECIES	COMMON NAME
Pinales	Cupressaceae	*Platycladus*	*P. orientalis*	Chinese Arborvitae
		Thujopsis	*T. dolabrata*	Thujopsis
		Chamaecyparis	*C. lawsoniana*	Lawson's Cypress
			C. obtusa	Hinoki Cypress
			C. pisifera	Sawara Cypress
		Juniperus	*J. chinensis*	Chinese Juniper
			J. virginiana	Eastern Juniper
			J. sabina	Savin Juniper
			J. phoenicea	Phoenicean Juniper
			J. communis	Common Juniper
			J. oxycedrus	Western Prickly Juniper
			J. excelsa	Greek Juniper
		Calocedrus	*C. decurrens*	California Incense-cedar
		Cupressus	*C. sempervirens*	Mediterranean Cypress
			C. sargentii	Sargent Cypress
			C. x leylandii	Leyland Cypress
		Cunninghamia	*C. lancelota*	China Fir
		Cryptomeria	*C. japonica*	Japanese Cedar$^{\#}$
		Thuja	*T. occidentalis*	Eastern Arborvitae
			T. standishii	Japanese Thuja
			T. koraiensis	Korean Thuja*
		Taxodium	*T. distichum*	Bald Cypress
		Metasequoia	*M. glyptostroboides*	Dawn Redwood
	Taxaceae	*Taxus*	*T. baccata*	European Yew
			T. cuspidata	Japanese Yew*
	Cephalotaxaceae	*Cephalotaxus*	*C. harringtonia*	Japanese Plum Yew
		Torreya	*T. californica*	California Torreya

Table S1. List of analyzed pollen taxa (part VI): gymnosperms. All samples collected in 2011 except (*) collected in 2012. All samples collected at the Botanical Garden of the Faculty of Science, except (#) collected at the Botanical Garden of the Faculty of Pharmacy and Biochemistry.

ORDER	FAMILY	GENUS	SPECIES	COMMON NAME
Pinales	Pinaceae	*Abies*	*A. koreana*	Korean Fir
			A. nordmanniana	Nordmann Fir*
			A. pinsapo	Spanish Fir
			A. cephalonica	Greek Fir
		Cedrus	*C. atlantica*	Atlas Cedar
		Picea	*P. abies*	Norway Spruce*
			P. asperata	Dragon Spruce*
			P. omorika	Serbian Spruce
			P. chihuahuana	Chihuahua Spruce*
			P. orientalis	Caucasian Spruce
			P. smithiana	Morinda Spruce*
			P. pungens	Blue Spruce#
		Tsuga	*T. canadensis*	Eastern Hemlock
		Pinus	*P. pinea*	Stone Pine
			P. mugo	Mountain Pine
			P. sylvestris	Scots Pine#
			P. tabuliformis	Chinese Red Pine
			P. armandii	Chinese White Pine*
			P. banksiana	Jack Pine
			P. pinaster	Maritime Pine#
			P. densiflora	Japanese Red Pine
			P. nigra	European Black Pine#
			P. ponderosa	Ponderosa Pine
			P. resinosa	Red Pine
			P. wallichiana	Himalayan pine
			P. bungeana	Lacebark Pine
			P. strobus	Eastern White Pine
			P. peuce	Macedonian Pine
			P. heldreichii	Bosnian Pine
	Podocarpaceae	*Podocarpus*	*P. neriifolius*	Brown Pine
Ginkgoales	Ginkgoaceae	*Ginkgo*	*G. biloba*	Ginkgo*
Ephedrales	Ephedraceae	*Ephedra*	*E. major*	Greater Ephedra

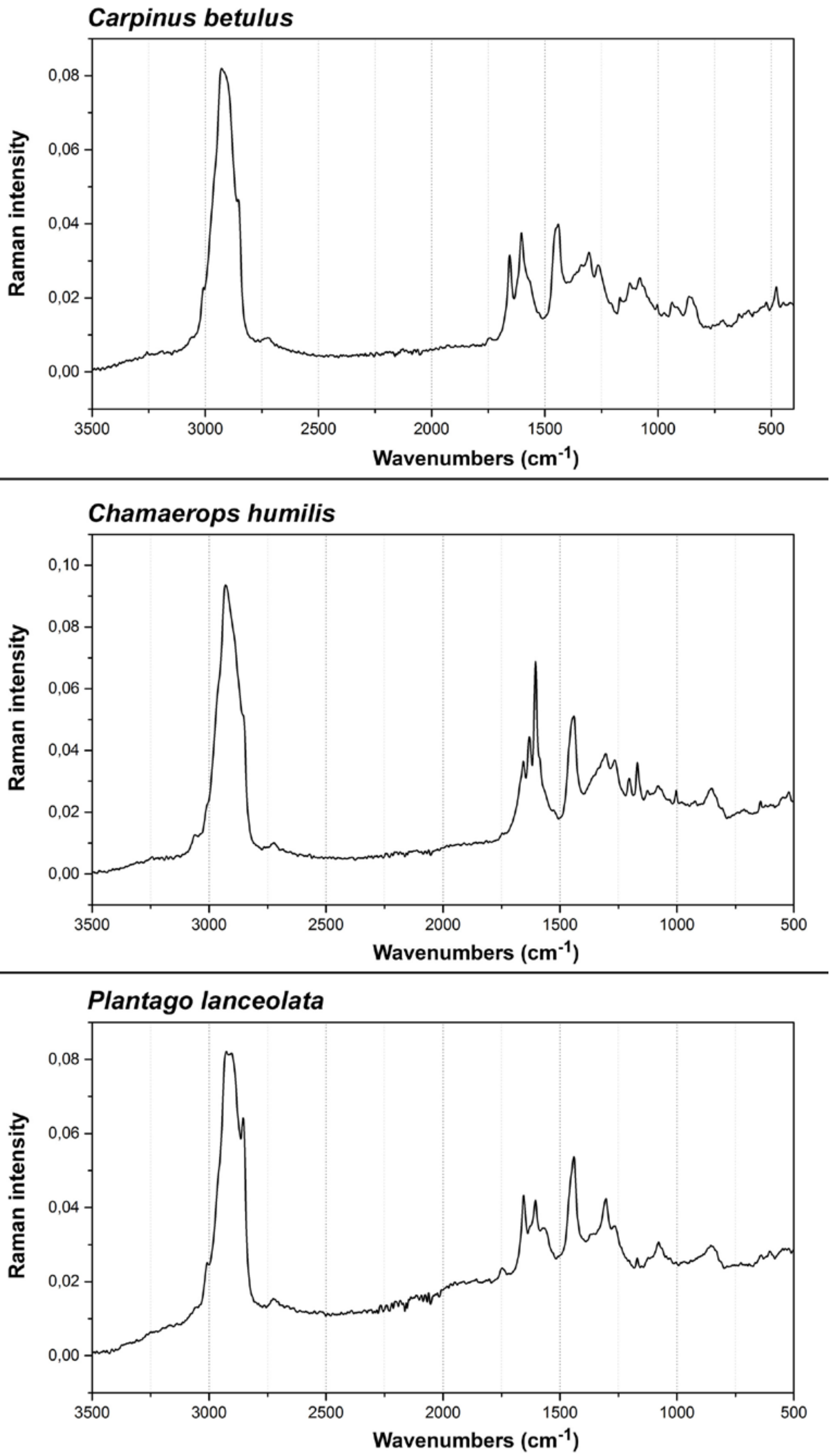

Figure S2. FT-Raman spectra of *Carpinus betulus*, *Chamaerops humilis* and *Plantago lanceolata* (see Figure S1 in Guedes et al. 2014).

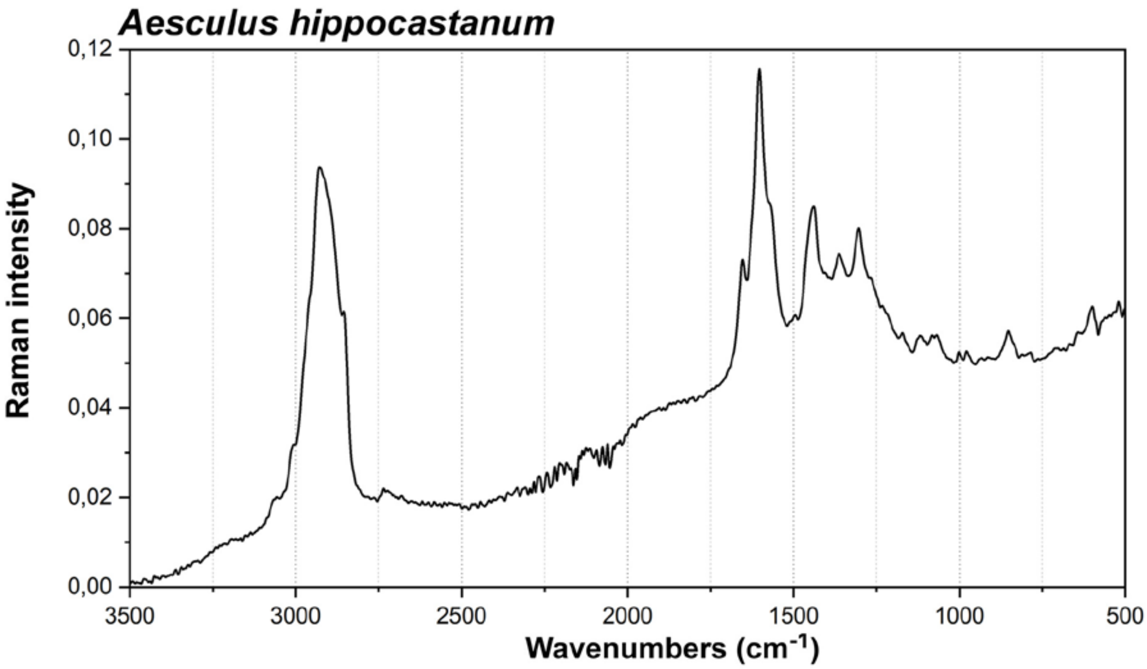

Figure S3. FT-Raman spectra of *Aesculus hippocastanum* (see Figure 1 in Schulte et al. 2008).

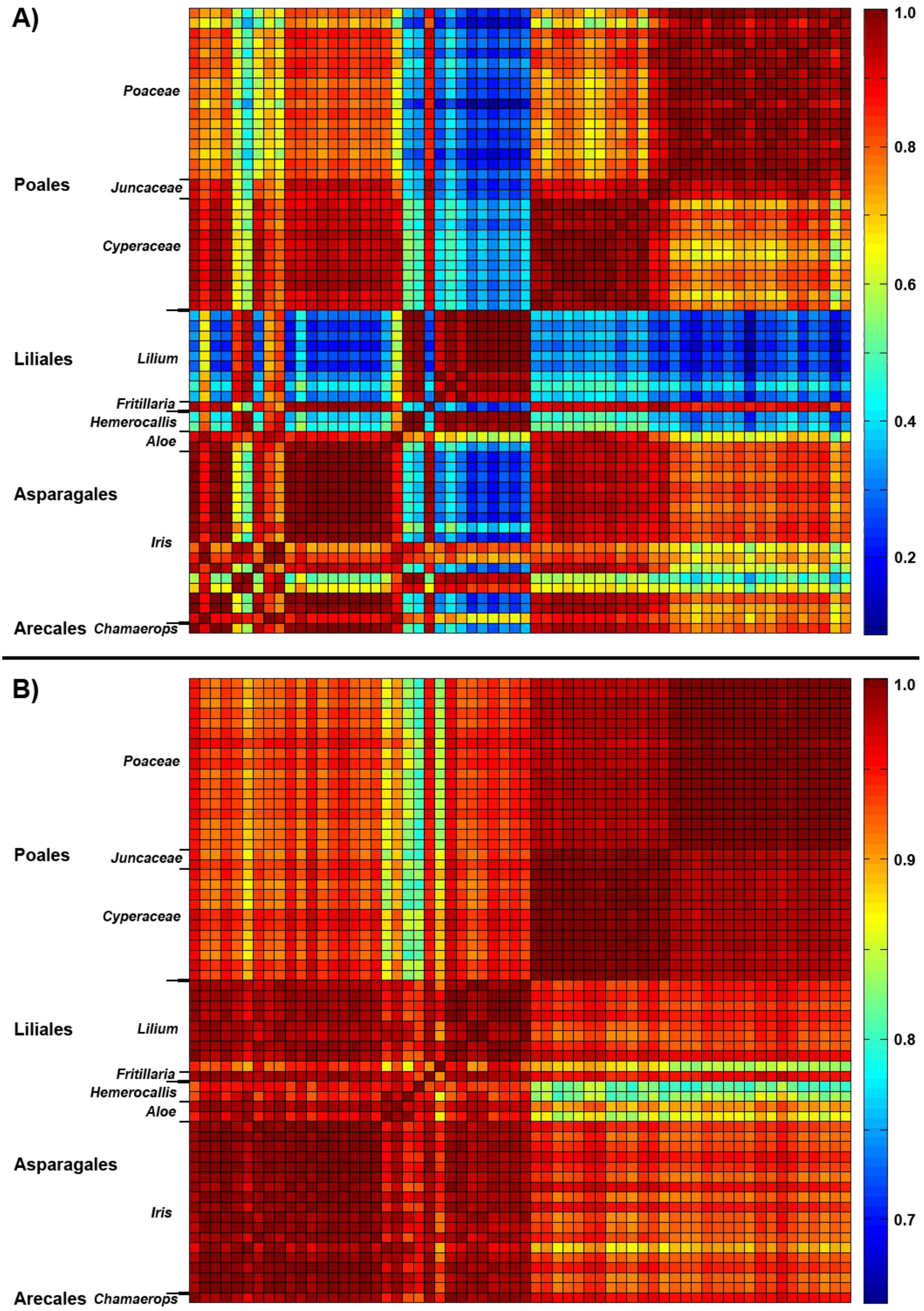

Figure S4. Matrices of correlation coefficients calculated from: (A) FT-Raman and (B) FTIR spectra of 63 monocots (Liliopsida species; average spectra of 3 measurements), with depiction of plant orders, families and genera.

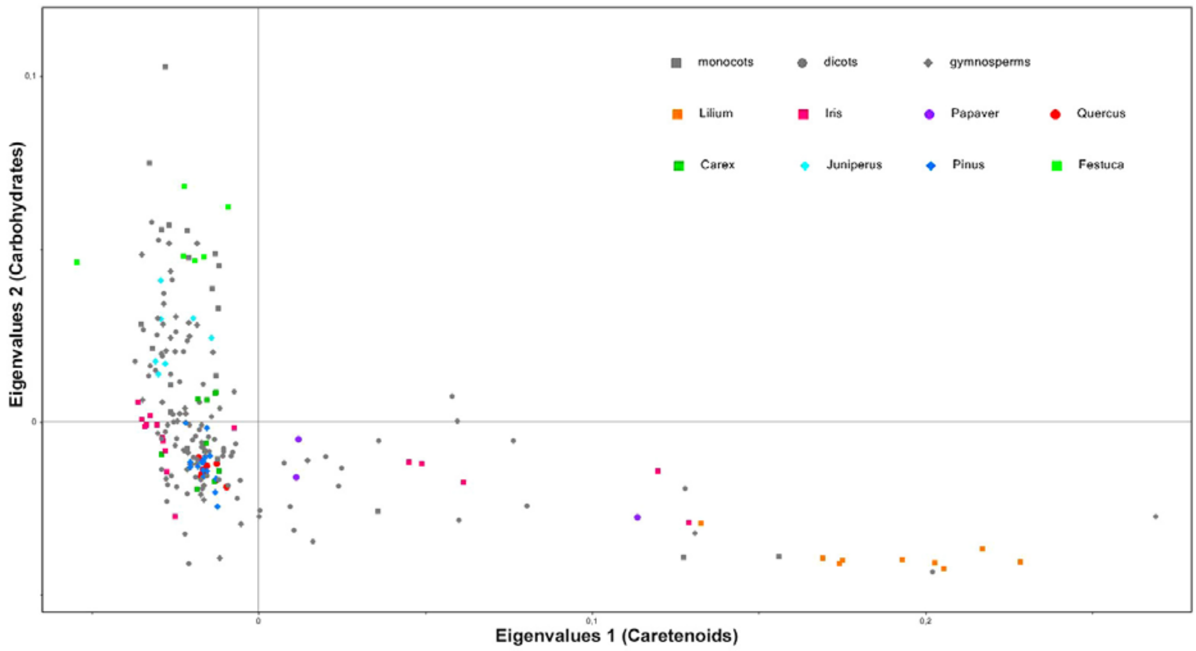

Fig. S5. Scatter plot of eigenvalues from the reduced FT-Raman data set (*Dataset I*, average spectra of 3 measurements), obtained by modelling spectral contribution of carbohydrates and carotenoids, with depiction of plant classes (■ monocots; ♦ gymnosperms; ● eudicots and magnoliids) and genera (*Iris*, *Lilium*, *Papaver*, *Quercus*, *Carex*, *Festuca*, *Juniperus*, and *Pinus*).

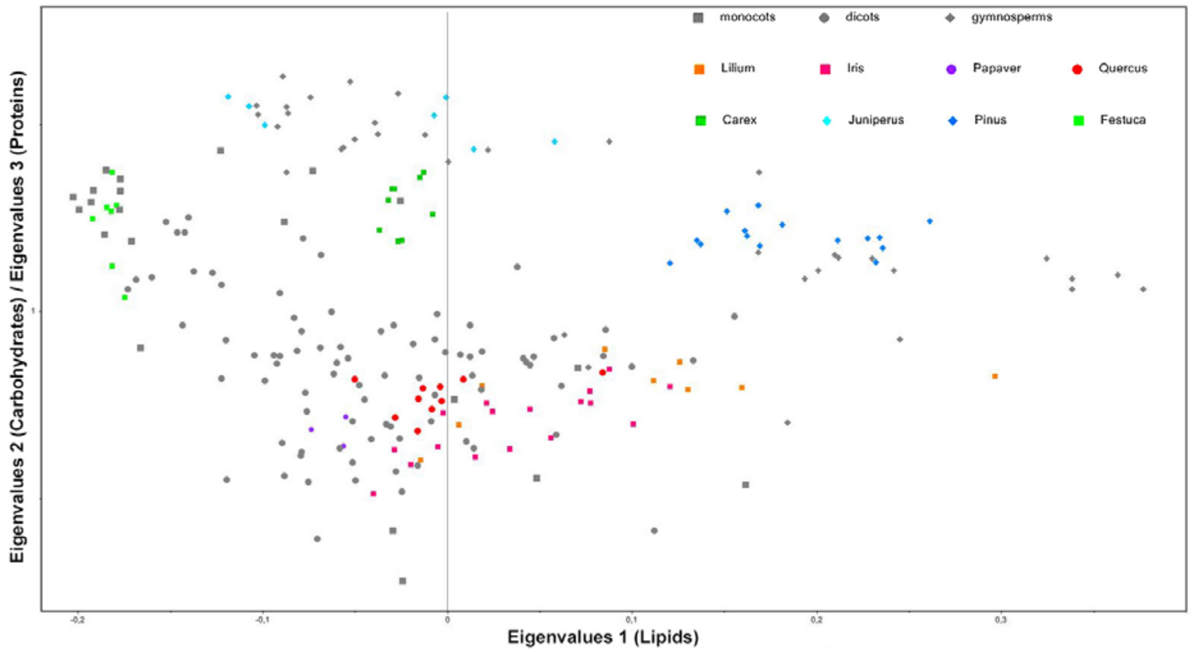

Fig. S6. Scatter plot of eigenvalues from the reduced FTIR data set (*Dataset I*, average spectra of 3 measurements), obtained by modelling spectral contribution of lipids, carbohydrates and proteins, with depiction of plant classes (■ monocots; ♦ gymnosperms; ● eudicots and magnoliids) and genera (*Iris*, *Lilium*, *Papaver*, *Quercus*, *Carex*, *Festuca*, *Juniperus*, and *Pinus*).

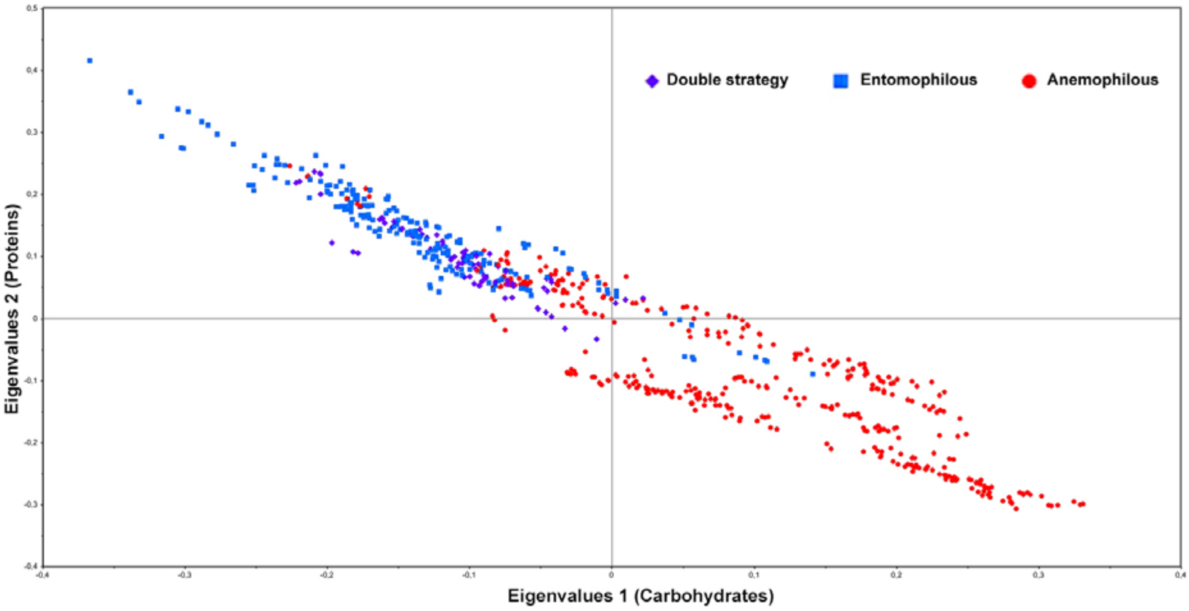

Fig. S7. Scatter plot of eigenvalues from the reduced FTIR data set (*Dataset I*, average spectra of 3 measurements), obtained by modelling spectral contribution of carbohydrates and proteins, with depiction of pollination strategies (blue■ entomophilous; red● anemophilous; purple♦ double-strategy) species.

Table S-2. List of 35 analyzed taxa for quantitative prediction of pollen protein content. The protein content of pollen from Roulston et al. (Roulston et al., 2000) was used as a chemical reference values for regression in the Partial Least Squares Regression (PLSR) modelling of spectral data from *Datasets I*.

Species	Protein content
Fagus sylvatica	17.4
Quercus rubra	40.6
Quercus robur	30.6
Corylus avellana	30.2
Alnus incana	23.5
Alnus glutinosa	24.2
Betula pendula	24.2
Juglans nigra	28.8
Juglans regia	23.6
Carya illinoinensis	25.1
Zea mays	20
Secale cereale	23.9
Festuca pratensis	24.6
Poa pratensis	24.6
Poa nemoralis	20.9
Dactylis glomerata	20.7
Holcus lanatus	25.6
Juniperus communis	22.2
Thuja occidentalis	8.8
Picea abies	9.8
Pinus mugo	20.9
Pinus sylvestris	13.7
Pinus ponderosa	15.7
Eschscholzia californica	11.7
Magnolia x sonlangiana	43.1
Liriodendron tulipifera	38.2
Fraxinus excelsior	37.1
Plantago lanceolata.	33.3
Salix alba	23.9
Taraxacum officinale	43
Populus nigra	36.5
Aesculus hippocastanum	26.7
Buxus sempervirens	29.5
Artemisia vulgaris	19.2
Rumex acetosa	19.5

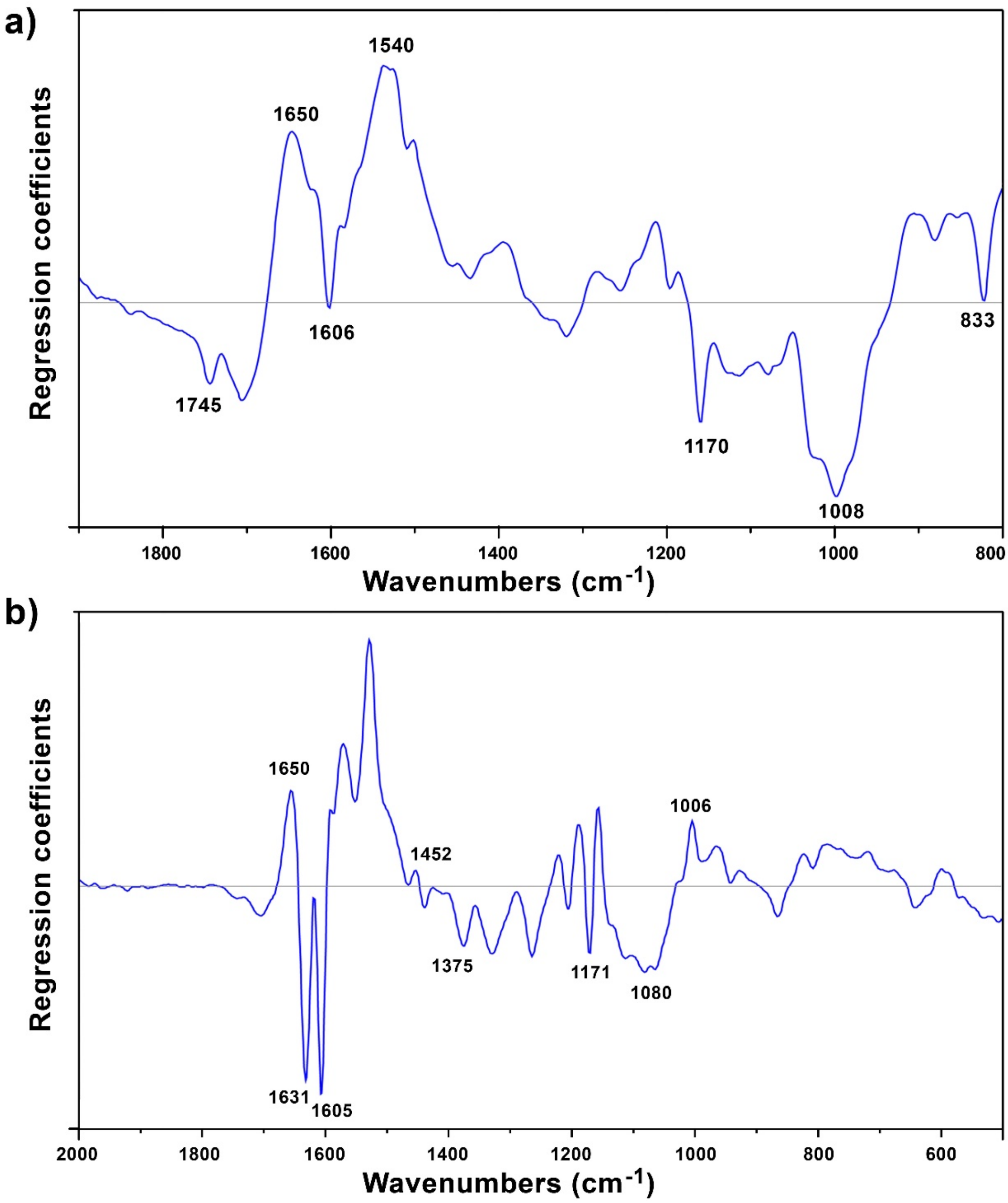

Fig. S8. PLS regression coefficients for: a) FTIR (the explained variances for factor 1: X-expl: 48%, Y-expl: 48%), and b) FT-Raman datasets (the explained variances for factor 1: X-expl: 41%, Y-expl: 17%). PLSR models are based on protein content for 35 pollen species (N = 35).

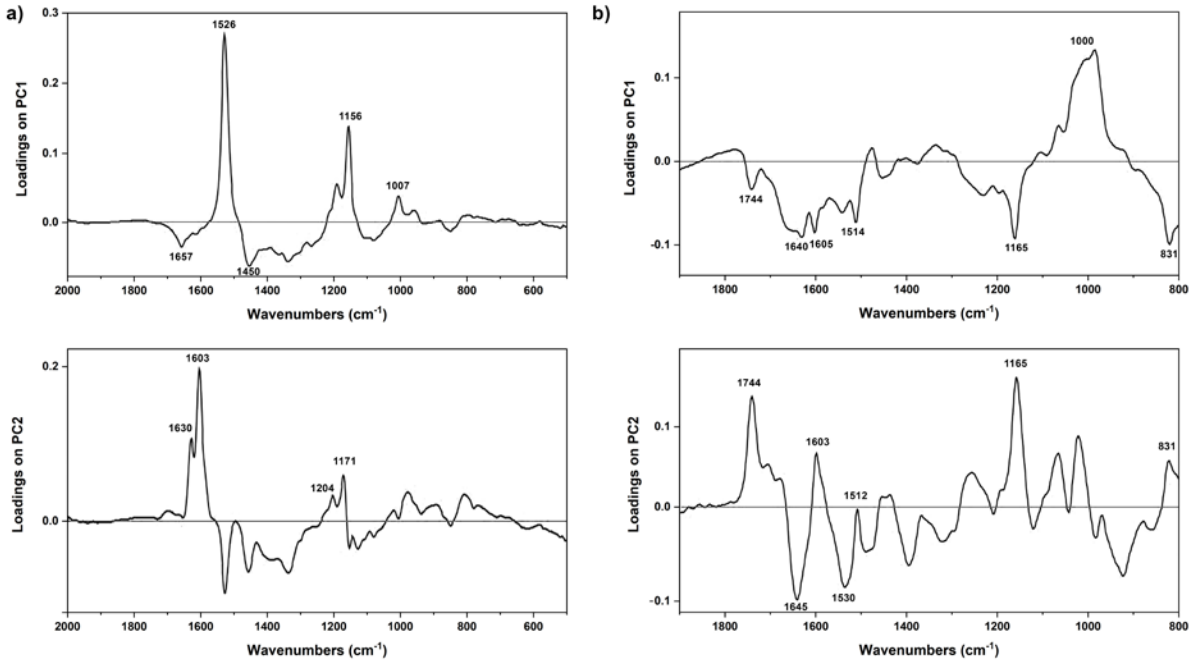

Fig. S9. Loadings plots on the first two principal components of a) FT-Raman and b) FTIR datasets for monocots (Liliopsida) (*Dataset I*, average spectra of 3 measurements) (see Figure 9. for score plots).

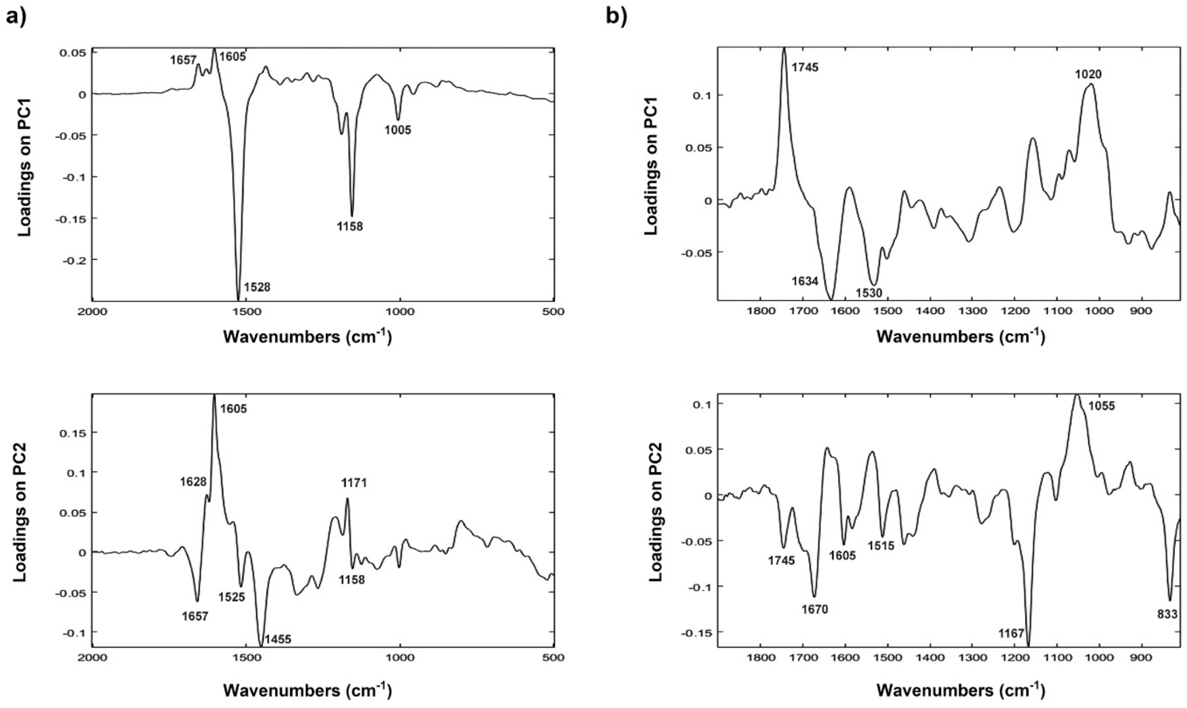

Fig. S10. Loadings plots on the first two principal components of a) FT-Raman and b) FTIR datasets for *Iris* (*Dataset I*, average spectra of 3 measurements) (see Figure 10. for score plots).

Table S-3. List of analyzed *Iris* taxa with depiction of floral traits. Relative lipid content is from Zimmermann and Kohler, 2014. Reference phylogeny data available for the indicated taxa (*) (Mavrodiev et al., 2014).

Subgenus	Species	Section	Series	Lipid content	Sepal color	Signal color	Pollen color	Nectar
Iris	*I. pallida subsp. illyrica*	*Iris*		124	purple	yellow	white	no
	I. pseudopallida	*Iris*		176	purple	yellow	white	no
	*I. pallida**	*Iris*		215	purple	yellow	white	no
	I. sikkimensis	*Pseudoregelia*		78	purple	white	white	no
Limniris	*I. versicolor**	*Limniris*	Laevigatae	113	purple	yellow	white	yes
	*I. pseudacorus**	*Limniris*	Laevigatae	64	yellow	yellow	yellow	no
	*I. sanguinea**	*Limniris*	Sibiricae	75	purple	yellow	white	no
	*I. sibirica**	*Limniris*	Sibiricae	52	purple	white	white	no
	*I. bulleyana**	*Limniris*	Sibiricae	50	purple	white	white	no
	*I. graminea**	*Limniris*	Spuriae	23	purple	yellow	orange	yes
	*I. spuria**	*Limniris*	Spuriae	76	purple	white	white	yes
	I. crocea	*Limniris*	Spuriae	33	white	yellow	orange	yes
	*I. orientalis**	*Limniris*	Spuriae	39	white	yellow	yellow	yes
	I. halophila	*Limniris*	Spuriae	33	white	yellow	orange	yes
	*I. unguicularis**	*Limniris*	Unguiculares	66	purple	white	white	no
	*I. japonica**	*Lophiris*		37	white	yellow	yellow	no
Scorpiris	*I. bucharica**	*Scorpiris*		77	white	yellow	white	no

Supplementary Material References:

Guedes, A., Ribeiro, H., Fernandez-Gonzalez, M., Aira, M.J. and Abreu, I. (2014) Pollen Raman spectra database: Application to the identification of airborne pollen. *Talanta*, 119, 473-478.

Schulte, F., Lingott, J., Panne, U. and Kneipp, J. (2008) Chemical Characterization and Classification of Pollen. *Anal Chem*, 80, 9551-9556

Roulston, T.H., Cane, J.H. and Buchmann, S.L. (2000) What governs protein content of pollen: Pollinator preferences, pollen-pistil interactions, or phylogeny? *Ecol Monogr*, 70, 617-643.

Zimmermann, B. and Kohler, A. (2014) Infrared spectroscopy of pollen identifies plant species and genus as well as environmental conditions. *Plos One*, 9, e95417.

Mavrodiev, E.V., Martinez-Azorin, M., Dranishnikov, P., and Crespo, M.B. (2014). At Least 23 Genera Instead of One: The Case of Iris L. s.l. (Iridaceae). *Plos One* 9.
